# Novel data analysis method for multicolour flow cytometry links variability of multiple markers on single cells to a clinical phenotype

**DOI:** 10.1038/s41598-017-05714-1

**Published:** 2017-07-14

**Authors:** Gerjen H. Tinnevelt, Marietta Kokla, Bart Hilvering, Selma van Staveren, Rita Folcarelli, Luzheng Xue, Andries C. Bloem, Leo Koenderman, Lutgarde M. C. Buydens, Jeroen J. Jansen

**Affiliations:** 1 Radboud University, Institute for Molecules and Materials, (Analytical Chemistry), P.O. Box 9010, 6500 GL Nijmegen, The Netherlands; 2 TI-COAST, Science Park 904, 1098 XH Amsterdam, The Netherlands; 3 Department of Respiratory Medicine and laboratory of translational immunology (LTI), University Medical Center Utrecht, Heidelberglaan 100, 3584CX Utrecht, The Netherlands; 4 Respiratory Medicine Unit, Nuffield Department of Medicine, University of Oxford, Oxford, OX3 7FZ United Kingdom; 5 Department of Immunology, University Medical Center, University of Utrecht, 3508GA Utrecht, The Netherlands

## Abstract

Multicolour Flow Cytometry (MFC) produces multidimensional analytical data on the quantitative expression of multiple markers on single cells. This data contains invaluable biomedical information on (1) the marker expressions per cell, (2) the variation in such expression across cells, (3) the variability of cell marker expression across samples that (4) may vary systematically between cells collected from donors and patients. Current conventional and even advanced data analysis methods for MFC data explore only a subset of these levels. The Discriminant Analysis of MultiAspect CYtometry (DAMACY) we present here provides a comprehensive view on health and disease responses by integrating all four levels. We validate DAMACY by using three distinct datasets: *in vivo* response of neutrophils evoked by systemic endotoxin challenge, the clonal response of leukocytes in bone marrow of acute myeloid leukaemia (AML) patients, and the complex immune response in blood of asthmatics. DAMACY provided good accuracy 91–100% in the discrimination between health and disease, on par with literature values. Additionally, the method provides figures that give insight into the marker expression and cell variability for more in-depth interpretation, that can benefit both physicians and biomedical researchers to better diagnose and monitor diseases that are reflected by changes in blood leukocytes.

## Introduction

Multicolour Flow Cytometry (MFC) is a powerful analytical technology that is used to measure (co)-expression of multiple markers at a single cell resolution. A typical sample for MFC analysis may contain large numbers of cells (>10000), characterized by the binding of several fluorescently labelled antibodies that represent expression of specific cellular markers^1^. The analysis of such marker (co)-expression has been essential in unravelling systematic and comprehensive patterns in *e.g*. haematopoiesis, cellular function and disease mechanisms^2^.

The information in MFC data is widely used for multiple objectives, the most prominent being: identifying cell subpopulations using two-dimensional gating and interpreting cell populations associated with a clinical phenotype^3^. Analysis of variability at the single cell level will provide clinically relevant information. Generally, MFC datasets contain information on four hierarchical levels, that range from the individual markers of the cell to the differential expression of functionalized cells in clinical phenotypes:

1. The multivariate (co)-expression of markers on single cells.
2. Aggregation of cells into populations with similar marker expression.
3. Representation of cell populations in a specific individual.
4. Differentiation of this representation in specific clinical phenotypes.

Several existing methods for MFC data analysis may predict accurately the phenotype a sample presents, but fail to quantitatively link the interplay between specific markers to *e.g*. disease specific cells^4^. Several methods use *e.g*. median fluorescence intensity or work with sequential two-dimensional gating steps to select specific cells. Two-dimensional multiple gating regions of interest are subjectively defined in bivariate plots, but important information is lost during every sequential two-dimensional gating step. Therefore, these methods can be viewed as multiple-univariate or multiple-bivariate and miss the comprehensive link between the four levels. Either approach does not employ the expression of more than two markers of a single cell, such that the MFC data is not interpreted to its full potential.

Recently, several multivariate methods have been developed that provide a canonical view on the (co)-expression of all markers. Fischer Information Nonparametric Embedding (FINE)^5^ uses Multidimensional Scaling to observe variability between samples, which may be used to explore response-related patterns. However, the link FINE draws between marker expression and variability between individuals is non-linear, such that relevant connections can only be drawn from *post hoc* interpretation of the model results. Likewise, the ViSNE^6^ approach visualizes the aggregation of cells for each individual by reducing the dimensions with a non-linear t-Distributed Stochastic Neighbour Embedding (t-SNE), such that the representation of single-cells (level 2) cannot be directly linked to the multivariate co-expression of markers (level 1). Moreover, neither method allows a projection of an individual sample (level 3) into the multidimensional model space to evaluate their similarity to samples in the calibration set on which the model was fitted (level 4). Several other methods that were specifically developed for MFC data analysis, use specific representations of the cellular composition of a sample (level 2) to find biomarkers for a clinical phenotype. Frequency Difference Gating^7^ (FDG), another method uses probability binning to get an equal number of cells in each bin, based on those measured in the control samples and then identifies bins differentially expressed in a specific (clinical) phenotype. The SPADE method constructs hierarchical clusters to differentiate cell populations^8^ that may be associated to a specific clinical phenotype^9^. Also Citrus^10^ uses hierarchical clustering to find such discriminating cell populations with an alternative regression approach called lasso-regularized logistic regression. Although these three approaches are multivariate and linear, they fail to show how the multivariate marker (co)-expression (level 1) underlies the cell diversity (level 2). Interpretations of such co-expressions are limited to qualitative comparisons between the expression of single markers in a multiple-univariate comparison.

Another common method in flow cytometry is Principal Component Analysis (PCA), which reduces the dimensions in order to study the most prominent variation in (co)-expression of all markers across all cells exhibited by specific clinical phenotypes^11, 12^. Two methods, Automated Population Separator (APS)^13^ and Flow cytometric Orthogonal Orientation for Diagnosis (FLOOD)^14^ use PCA. The advantage of PCA is that you can represent the single cells together with the interrelated expressions of the markers in a biplot. The APS method creates a PCA model for every phenotype based on all the cells of the individuals in that phenotype. Next, a new measurement is projected into each PCA model and the closest clinical phenotype is selected as the predicted phenotype. FLOOD builds a PCA model based on the marker variability in healthy individuals and subsequently models the variability only observed in responding individuals. FLOOD then counts the number of responding cells. However, some cells may be more specific for a clinical phenotype than others. Therefore, it would be better to use regression to give specific cells higher weights.

In this paper we present Discriminant Analysis of MultiAspect CYtometry (DAMACY), a multivariate method that uses PCA biplots and multivariate regression based on Partial Least Squares (PLS) to quantitatively compare the leukocyte compositions of multiple individuals, specifically those correlated to the discrimination of individual groups based on their immune response. Thereby, DAMACY can integrate all four levels of MFC information and in this way, differentiate between clinical phenotypes based on quantified co-expression of multiple markers on different cell populations.

**Table 1:** Expression profile of the different areas marked with symbols in Fig. 5. The expression profiles are checked by conventional sequential two dimensional gating.

Symbol	Expression	Cell type	Proportionally more represented in:
■	CD3+CD8−CD4++	CD4 T cell	Asthma
□	CD3+CD8−CD4+	CD4 T cell	Control
▲	CD3+CD4−CD8++	CD8 T cell	Asthma
△	CD3+CD4−CD8+	CD8 T cell	Control
♠	CD3+CD8+CD4+	Double Positive T cells	Asthma
◇	CD3+CD4−CD8−	Double Negative T cells	Control
⌂	CD14+	Monocyte	Control
▼	CD16+	Neutrophil	Asthma
origin	CD16_med_	Neutrophil	Control (median cell)
▽	CD16_dim_	Neutrophil	Asthma
▶	CD16_dim_CRTH2+	Eosinophil	Asthma
☆	low expression	Debris/dead Cells	Control

## Results

We demonstrate the strength that DAMACY has to describe homeostasis and deviations in the haematological and immunological system by three complementary case studies. We acquired the first dataset to study the *in vivo* effect of lipopolysaccharide (LPS) on blood neutrophils. The second data set focussed on the identification of tumour cells within bone marrow samples of patients with acute myeloid leukaemia (AML). We obtained the third dataset from total leukocytes isolated from patients with different phenotypes of asthma. The control group in this article is stated as controls and the other group as challenged individuals.

For benchmarking against other methods, we evaluated the predictive ability of DAMACY in two Flow Cytometry: Critical Assessment of Population Identification Methods (FLOWCAP) II challenge datasets^4^. The 99% accuracy on the AML and 100% accuracy on the HIV Vaccine Trials Network (HVTN) datasets of DAMACY is comparable to the other evaluated methods. We provide more details in the Supplementary material.

### Lipopolysaccharide challenge

Intravenous administration of lipopolysaccharide (endotoxin) elicits a systemic inflammatory response syndrome in humans (SIRS)^15^. Neutrophils demonstrate a characteristic difference in expression of CD16 and CD62L, together with a larger array of alterations in co-expressed surface markers^15^.

**Figure 1:**
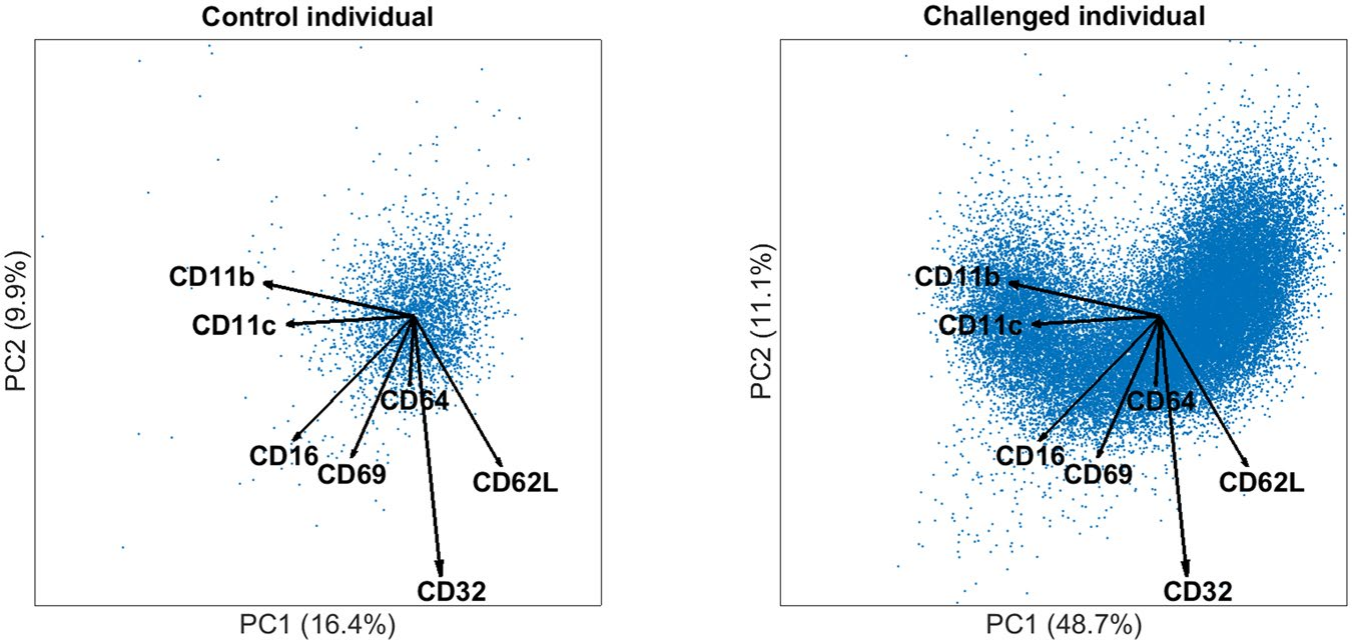
Shows a biplot of the cells of a typical control (left) and a typical LPS-responding individual (right). Each dot represents a score, thus a single cell. The model loadings are represented by vectors and indicate how each surface marker contributes to the cell variability in a specific direction. The percentage given is the explained variance per PC of that individual.

**Figure 2:**
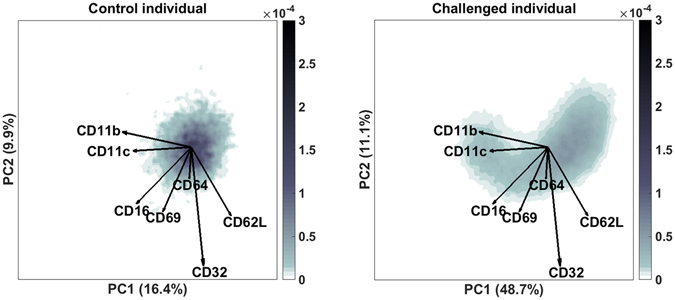
Smoothed histograms of the biplots shown in Fig. 1 of a typical control (left) and a typical LPS-responding individual (right). The darker bin, the more cells are likely present in that location. The same loadings are plotted on top as vectors and indicate how each surface marker contributes to the cell variability in a specific direction.

The DAMACY model on the LPS data (Fig. 1) required two Base Principal Components (PCs) to explain 65% of the total variability across cells within all samples. Specifically, the first PC explains considerably more variability in challenged individuals (59%) than in controls (19%), which indicates that the Base model describes considerable response-specific cell variability. The score distributions show that this response entails a distinctive ‘bean’ shape following a continuous gradient in marker expression (see figure right panel Fig. 2) that is not observed in control individuals.

Qualitative comparison of both biplots show clear differences induced by LPS. The individual cell scores can however not be directly quantitatively compared between individuals: such comparison needs to transcend the single-cell level. This could be achieved by gating the cells, however the observed continuous distribution in marker expression prohibits setting a discrete gate. Therefore, we used a small binsize to bin the cell scores in order to retain as much resolution in the marker expression as possible. Supplementary Figure 1 shows the cellular distribution of a typical sample in an unsmoothed histogram, which is very grainy and therefore poorly comparable between samples. Histogram smoothing resolves the continuous distribution that underlies these bins, which can be much better quantitatively compared between samples (Fig. 2).

**Figure 3:**
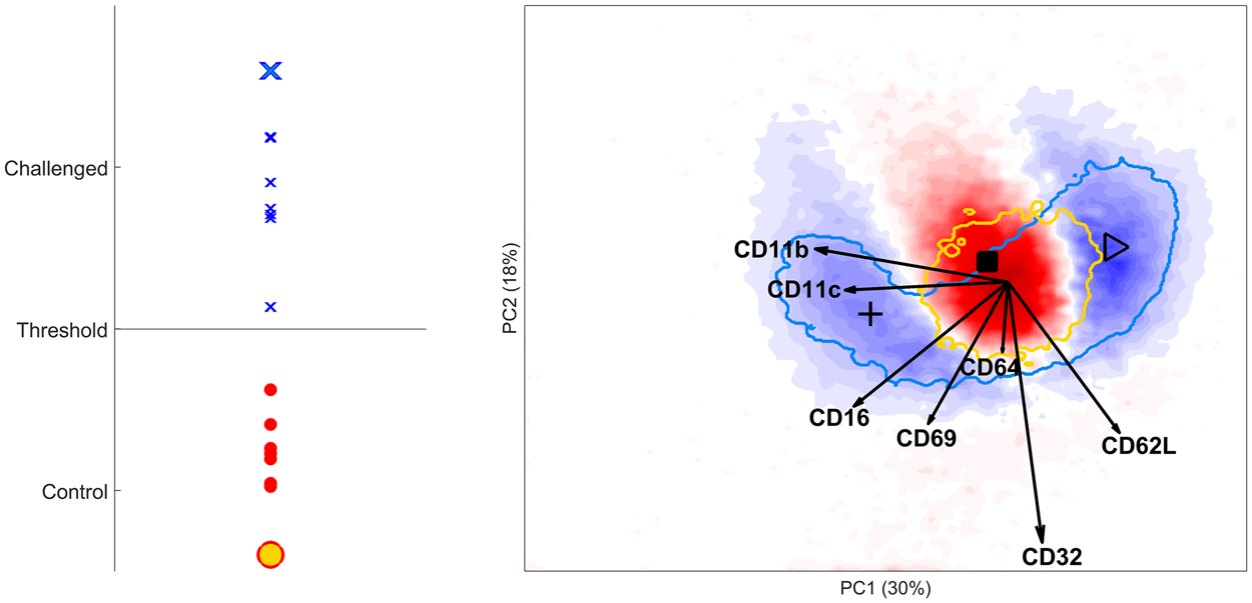
DAMACY model of LPS data. The left panel shows the average prediction score of controls (red rounds) and LPS-responding individuals (blue crosses) based on the results of the double cross-validation. The right panel shows negative weights as red and positive weights as blue. The blue contour depicts where most cells of the enlarged cross are. The red contour depicts where most cells of the enlarged round are. The loadings of the Base model are plotted on top as black vectors and indicate how each surface marker contributes to the cell variability in a specific direction. The marker expression of cells near ▷, ■, + are discussed in the text.

The immune response of neutrophils to LPS, can be interpreted by quantitative comparison of these smoothed histograms. We performed this comparison by OLPS-DA, which leads to perfect diagnosis of the LPS-specific response (Fig. 3, right). The blue and red regions indicate the most discriminative bins, coloured according to their weight in the discriminant model, where blue bins contain a larger fraction of cells in challenged and red bins larger fractions in the control samples. The cross-validated prediction scores (Fig. 3, left) show the classification of each sample as either challenged or control based on these weights.

Two benchmarks, coloured in orange and blue in Fig. 3, encircle the 80% bins most highly occupied by single cells. The area with the most decreasing cell fraction decreases upon LPS response (coloured most intensely red in Fig. 3) is also encircled by the blue benchmark, which indicates that peripheral blood of responders contains a considerable fraction of cells with this surface marker expression, identified as normal mature neutrophils, even upon response^16^.

The most intensively in- or decreasing bins in the leukocyte map may be subdivided into three distinct regions that we gated for further inspection (Supplementary Figures 2 and 3). Control samples contain a higher fraction of cells near ■ than responders. These cells have an average expression of all markers. Cells near + are more abundant in LPS responders; their expression of CD11b, CD11c, CD16 and CD69 is higher. Cells near ▷ are also more abundant in LPS responders, but the expression of CD11b, CD11c, CD16 and CD69, is lower than average while they co-express more CD62L. The elongated shape of the blue coloured bins near ▷ along the CD62L loading shows that cells vary considerably in their expression of this marker. The expression of CD32 and CD64 is not oriented towards any specific region, and therefore does not associate with specific groups of cells responding to LPS. The large loading of CD32 indicates that the expression of this marker varies considerably between all neutrophils. The small CD64 loading shows it does not contribute to the modelled marker expressions.

The continuum in marker expression could not be observed in the bivariate plot of the most prominent surface markers CD62L and CD16 (Supplementary Figure 4). Rather, these two markers alone show a fully independent and orthogonal behaviour for all cells. However, when observing the linear relationships between all markers simultaneously in DAMACY, a much more gradual continuum is observed. The median fluorescence intensity of the cells within the three gated regions (Supplementary Figure 3) shows that considerable overlap exists in single marker expression between the gated regions. Thus, the multivariate marker co-expressions provide a much more distinctive view on the immune response to LPS than single-marker evaluation of the same data.

### Acute Myeloid leukaemia

AML is characterised by an expansion of abnormal immature myeloid cells in both the bone marrow and peripheral blood. The cellular composition of 15 bone marrow samples was analysed by MFC using the EUROFLOW ALOT screening approach^17^. Nine bone marrow samples were from AML patients and six from individuals with absence of any leukemic clones in the bone marrow (controls).

**Figure 4:**
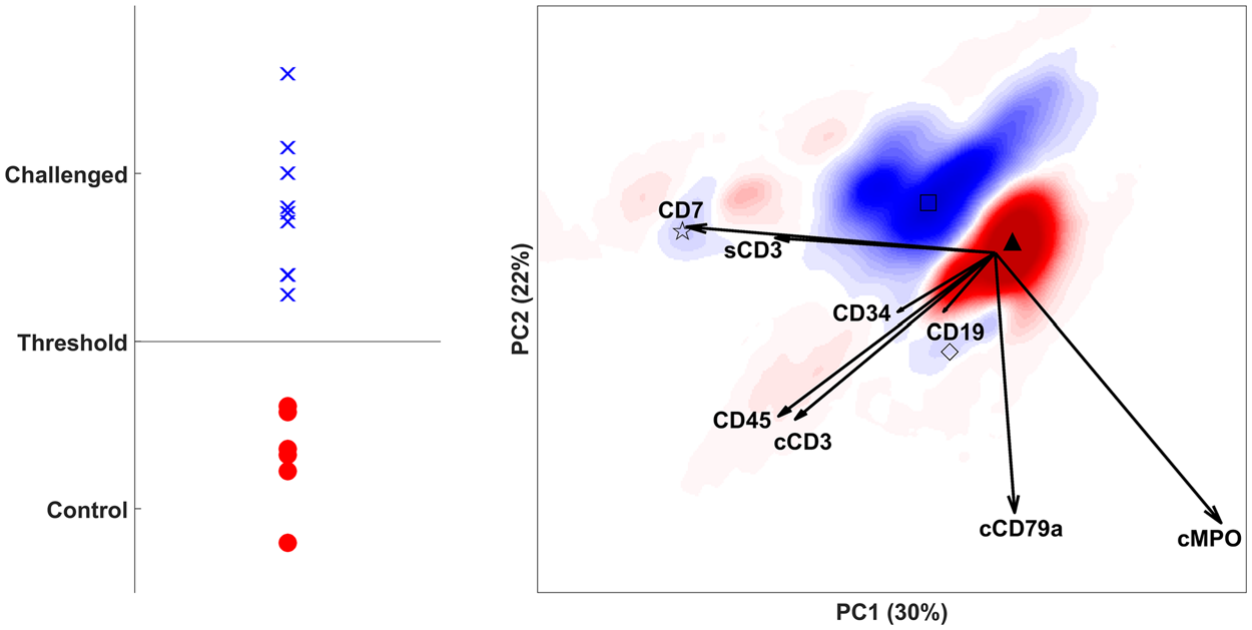
DAMACY model of AML data. The left panel shows the average prediction score of controls (red rounds) and AML patients (blue crosses). The right panel shows negative weights as red and positive weights as blue. The loadings of the Base model are plotted on top as black vectors and indicate how each marker contributes to the cell variability in a specific direction.

DAMACY analysis correctly discriminated between controls and AML patients (Fig. 4) with 98% accuracy in a six-fold cross leave-sample-out validation with 20 iterations^10, 18, 19^. The haematological map (right panel) shows an intense red region near ▲ with cells that are underrepresented in AML patients and an intense blue region near □ with cells that are overrepresented in AML. The cells near ▲ have a higher expression of cMPO compared to cells near □; cells in both regions are myeloid because they have average or high expression of cMPO and low expression of other markers. Another, less intensely blue region near ◇ contains B-cells with higher expression of CD19 and cCD79a. The T-cell populations position near ☆, with relatively high expressions of CD7 and sCD3. These latter populations are coloured less-intensely blue and are therefore decreasingly important in the observed AML. DAMACY may show specific AML responses, namely a higher amount of myeloid cells and not in T cells or B cells. An additional finding is the lower expression of cMPO in myeloid cells in AML individuals.

### Asthma phenotypes

Asthma is a heterogeneous disease characterised by symptoms of wheezing, cough, breathlessness and chest tightness. Current diagnosis is based on symptoms and variable airflow limitation^20^. In the current analysis we compared 24 asthma patients to 10 healthy controls. Their blood samples were characterized by eight antibodies: CD3, CD4, CD8, CD14, CD16, CD123, CD193 and CD294 (CRTH2). This surface marker panel specifically differentiates between asthma-associated cell types, such as eosinophils, type 2 T helper cells (Th2-cells), type 2 cytotoxic T cells (Tc2) and basophils. The CRTH2-antibody was included to measure the presence of the PGD2 receptor^21–23^.

Each individual measurement was centred in the Base model by median based on the whole dataset, as this led to highest prediction accuracy among all evaluated methods. Systematically evaluation of Top models based on all combinations of these Base components showed that histograms of components 1 and 3 provided 91% accuracy in discriminating mild/severe asthma patients versus controls.

**Figure 5:**
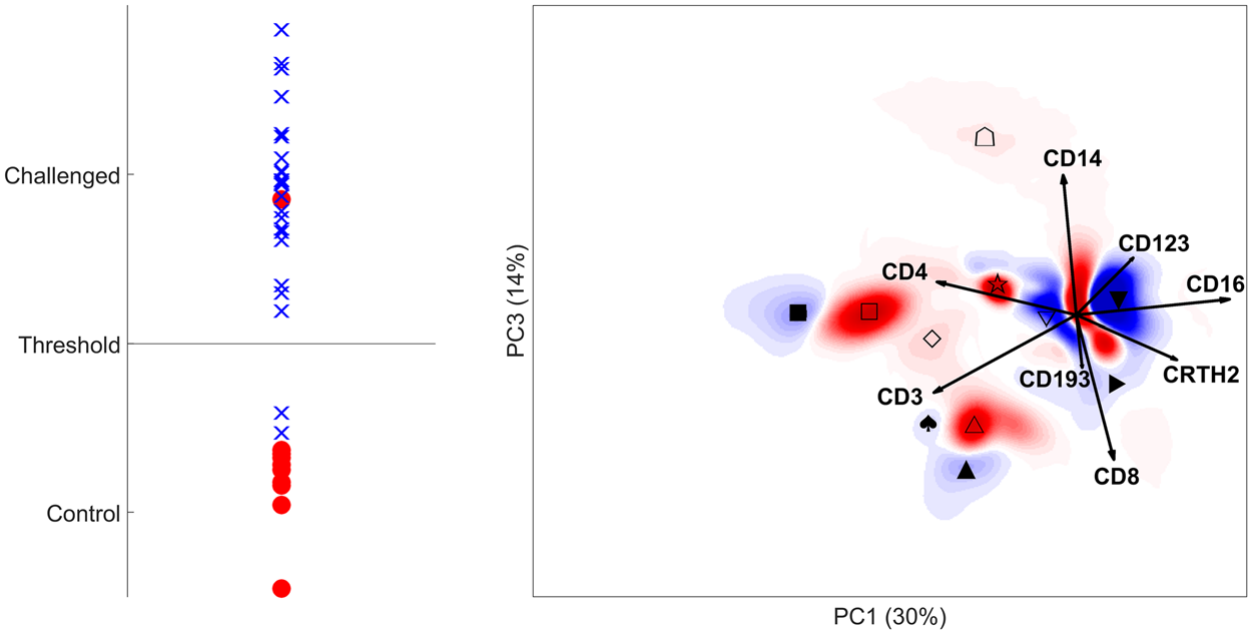
DAMACY model of asthma data. The left panel shows the average prediction score of the OPLS-DA model of controls as red rounds and asthma individuals as blue crosses. The right panel shows negative weights as red and positive weights as blue. The loadings of the Base model are plotted on top as black vectors and indicate how each surface marker contributes to the cell variability in a specific direction within the model. The symbols indicate the centroid of that coloured area that are representative for different cell populations (see Table 1).

The OPLS-DA model (Fig. 5 right panel) shows the leukocyte map. Many different coloured regions can be found in Fig. 5 where the centroid point is denoted with a symbol. The marker expression profiles of the areas are given in Table 1. In the origin are the neutrophils which is likely due to median centering with neutrophils as the most common cell type. The neutrophil with average CD16 expression is more represented in controls, while the neutrophils with higher CD16 expression and with lower expression are more represented in asthma patients. This CD16 pattern was also seen in the LPS data. In asthma patients the CD3+CD4+ T cells express more CD4 and the CD3+CD8+ T cells more CD8 compared to control individuals. Naturally, asthma patients have more eosinophils. Monocytes and CD4/CD8 double negative T cells on the other hand are slightly more represented in controls. Basophils are not correlated to any of the two groups. Th2 cells and Tc2 cells overlap with the CD4 and CD8 cells, respectively. The leukocyte map gives, in one overview, the cell types that are more represented in asthma patients.

**Table 2:** The four information levels of multidimensional flow cytometric data.

Information level	DAMACY output
(1) The multivariate (co)-expression of markers on single cells	PCA (loadings)
(2) Aggregation of cells into populations with similar marker expression	PCA (scores)
(3) Representation of cell populations in a specific individual	Histograms
(4) Differentiation of this representation in specific clinical phenotypes	Leukocyte map based on OPLS-DA weights.

## Discussion

The systematic integration of MFC data into four distinct levels (see Table 2) can be quantitatively linked together to improve the interpretation of comprehensive cell variability in marker co-expression. DAMACY combines multivariate ordination and classification of markers, cells, individuals and clinical phenotypes. The four levels provide adequate diagnoses in our complementary case studies and link this to individualized responses in single cells and in co-expression of several markers.

For example, LPS leads to emergence of cells reduced in their expression of CD62L and other cells considerably reduced in CD16 expression (Fig. 3). Cells which highly express CD16 and lowly express CD62L are also high in expression of CD11b and CD11c, a **relationship between surface marker expressions** that can be directly interpreted from the Base loadings within the leukocyte map. These cells are absent in homeostasis and can be used as diagnostic for acute inflammation.

The histograms show that rather than discrete populations, the neutrophil compartment of LPS responding individuals **contains cells with a continuum** of co-expression levels of these markers that cannot be observed in the separate expression levels. Relative differences in expression throughout this entire continuum may be distinct between groups of individuals, which may not be observed in SPADE and Citrus that rely on presence of discrete populations/clusters. To alleviate this, SPADE and Citrus use hierarchical clustering with many clusters in order to model the continuum. However, too many clusters results in difficulties in interpretation, while too few clusters may not capture all the information present. Because DAMACY does not use clustering but PCA, the clusters or continuum will appear as is present in the data, with easy to interpret figures as the vector loadings show the relative marker expression. **Individualized aspects of this expression** can be visualised within the DAMACY framework by superimposing cell variability within an individual sample onto the leukocyte cell map. The latter shows that this variability indeed involves up-regulation across the entire continuum for a specific individual (Fig. 3). The predictive scores, finally, quantify the difference between both groups of individuals, and allow a **robust, comprehensively statistically validated result**. These results show higher percentages of cells that are simultaneously low in CD62L and high in CD11b are indicative of endotoxemia, although the expression of these markers may vary across cells. These cells form a relatively larger proportion of the neutrophil population in some challenged individuals than in others; clearly there is considerable heterogeneity across all four levels that may be interpreted by DAMACY.

Amir *et al*. have described that single-cell data has many non-linear relationships, which cannot be used by a linear method such as PCA^6^. Non-linear relationships do, however, not disappear in linear models, as the biplot representation of the cell variability in LPS shows (Figs 2 and 3) a bean-like shape due to that healthy cells have a high expression of both CD16 and CD62L highly, whereas cells responding to LPS either express CD16 or CD62L highly. These effects were also observed with FLOOD^14^. However, FLOOD looks for response specific cells per individual, while DAMACY focuses more on the ratio of specific cells between controls and challenged individuals and gives a general leukocyte map for all challenged individuals. Non-linear methods such as ViSNE^6^ and FINE^5^ have the drawback that the quantitative view on marker co-expressions is lost from the model. Also linear clustering methods like SPADE^8^ and Citrus^10^ allow only qualitative interpretation of marker co-expression, either through similarities between heatmaps of individual markers or by plotting the histograms of the individual markers of a single cluster as is done in Citrus and in the non-linear binning of FDG^7^. These methods thus fail to quantitatively connect the variability in marker (co)-expression on single cells with the ratio between these cells between MFC samples of different phenotypes.

### The clinical value of DAMACY

The information content within MFC data revealed in a quantitative model strongly depends on the specific choices made in collecting the data and in data processing that precedes modelling. The analysed sample may consist of a relatively homogeneous cell population or can interrogate a much broader mixture of cells, such as the whole blood and bone marrow analysed in the asthma and AML studies presented here, respectively. The results on the FLOWCAP 2 data show how the quantitative potential of DAMACY is on par with the most advanced methods benchmarked in this challenge. The discriminatory Top model may extract the response-related information on cell variability in marker expression from the Base model histograms, both for *a priori* gated homogeneous neutrophil populations (LPS) and in peripheral blood (asthma). DAMACY shows to be also very informative for other sample types, like bone marrow samples from the AML study.

Diagnostic ability is however not the only, and certainly not the most challenging, issue in current MFC data analysis. The challenge is finding *all* variability in biological samples that is relevant to the studied response and thus disregarding all irrelevant variability. This implies that markers can be disregarded in the future leading to more focused experiments. The asthma dataset contains some older asthma patients outside the confidence interval of the age of the controls. The current model is unable to separate the variance caused by age from the disease and this would be possible if future experiments contain age matched controls. In that way, the method will minimize the variance explained caused by age and maximize the variance caused by the disease.

Cell distributions, identified by the Base model histograms, may vary greatly across individuals, and even systematically in aspects that do not align with the studied classification. An unsupervised PCA-based Top model may be very helpful for such observations (Supplementary). In the LPS study this model reveals a dim/bright marker profile that varies systematically among individuals in both groups, which therefore remains enveloped in the OPLS-DA Top model. Also the superimposing of individual histograms (Fig. 3) may be a key advantage of DAMACY for this.

## Conclusion

The DAMACY method can be used to objectively discriminate specific clinical phenotypes and provides a completely statistically validated model that proves insightful in a range of case studies. The method does not require any input on cell characteristics relevant to the experiment and is thereby unbiased to the prior knowledge of the researcher. The DAMACY model is able to quantitatively link individual variability in a clinical phenotype to specific cell populations with typical levels of marker co-expression that provide a novel, broad view on responses in health and immunological/haematological diseases.

## Methods

### Data

Subjects with asthma and normal subjects participating in the LPS challenge study gave peripheral blood. All data were obtained by using standardized protocols. All studies and sample collection were approved by the medical ethics committees of University Medical Centers Utrecht (asthma patients/UMCU) and Radboud University Medical Center (LPS challenge/RadboudUMC), the Netherlands. These participants gave their written informed consent. AML data were from bone marrow and were extracted from Anonymized flow cytometry list mode data sets (fcs files). These data sets were originally acquired as part of the UMCUtrecht institutional work up scheme for evaluation of leukaemia.

### Acute myeloid leukaemia data (AML)

Bone marrow samples were analysed for the presence of AML by MFC for diagnostical purposes using the acute leukemia orientation tube (ALOT) as described by the EuroFlow consortium^17^. The ALOT tube is a single 8-color-tube designed for the identification of an expanded population of immature blast like cells, including AML blasts. The 8-color panel consists of detection of CD3 (cytoplasmic and surface), MPO (cytoplasmic), CD79a (cytoplasmic) and the surface expression of CD19, CD34, CD45 and CD7. The uncompensated list mode fcs data files of bone marrow samples (100.000 stained cells) of 9 AML patients and 6 individuals where no haematological malignancy could be detected in the BM, were used in this study. The flowcytometric analyses were performed on a FACSCanto II (Becton Dickinson).

### Lipopolysaccharide (LPS) challenge

In the LPS challenge dataset there are 16 individuals: 8 healthy controls and 8 that have undertaken the LPS challenge. Flow Cytometry measurements were performed during an endotoxin trial (NCT01374711; www.clinicaltrials.gov). Details regarding the Flow Cytometry experiments that provided the data to illustrate the method are described in the Online Supplement I; source data can be accessed through www.flowrepository.org ID: FR-FCM-ZZEE. This dataset has been extensively analysed in two earlier publications.

### Asthma

The asthma dataset contains 24 asthma patients (aged 22–78, $\overline{{\rm{x}}}=57$) and 10 healthy controls (aged 25–57, $\overline{{\rm{x}}}=40$) were recruited at the respiratory outpatient clinics of the Churchill Oxford University Hospital, UK. The study received ethical approval, and written informed consent was obtained. After inclusion patients filled out symptom questionnaires, sputum induction was performed, blood was taken, and patients underwent FeNO measurement and lung functional testing. All patients were receiving appropriate asthma treatment at the time of blood withdrawal. Blood cells were stained with a panel of 8 antibodies including CD3, CD4, CD8, CD14, CD16, CRTH2 (CD294), CD123 and CD193. After staining, red blood cells were lysed using a FACS Lysing solution (Becton Dickinson). Cells were measured on a LSR Fortessa flow cytometer (Becton Dickinson).

MFC data can be arranged into matrix $${\bf{X}}=\,[\begin{array}{c}{{\bf{X}}}_{{1}_{1}}\\ \vdots \\ {{\bf{X}}}_{{I}_{G}}\end{array}]$$ of size $\,(\sum _{{i}_{g}={1}_{1}}^{{I}_{G}}{N}_{{i}_{G}}\times \,J)$ for analysis by DAMACY, where ${N}_{{i}_{g}}$ is the number of cells per individual *i*_*g*_ = 1_1_, … *I*_1_, … 1_*G*_, … *I*_*G*_; *g* = 1, ….. *G* indicates the pre-defined groups of individuals with *g* = 1 representing the control group and *g* = 2 the patient/challenged group, with *j* = 1, …, *J* indicates the markers. The data of each individual can analogously be denoted as

$${{\bf{X}}}_{{i}_{g}}=[\begin{array}{c}{{\bf{x}}}_{{1}_{{i}_{g}}}^{T}\\ \vdots \\ {{\bf{x}}}_{{N}_{{i}_{g}}}^{T}\end{array}]\,\,$$

of size $({N}_{{i}_{g}}\times J)$ and the data of each class as

$${{\bf{X}}}_{g}=[\begin{array}{c}{{\bf{X}}}_{{1}_{g}}\\ \vdots \\ {{\bf{X}}}_{{I}_{g}}\end{array}]\,{\rm{of}}\,{\rm{size}}\,(\sum _{{i}_{g}={1}_{g}}^{{I}_{g}}{N}_{{i}_{g}}\times \,J).$$

### Stage 1: Data pre-processing

Data pre-processing is used in multivariate analysis, to remove artefacts and thereby enhance the biologically relevant information^24^. First, we log-transformed the matrix **X** to **X**_log_ = log_10_(**X**)or in the case of negative values we prior subtracted the minimum value. Then we centred the data, to create a common point of reference to quantify the *variability* in marker expression between all cells.

1A $${{\bf{m}}}_{{i}_{g}}^{{\rm{T}}}=\,\frac{{\sum }_{n=1}^{{N}_{{i}_{g}}}{{\bf{X}}}_{{\mathrm{log}}_{{i}_{g}}}}{{N}_{{i}_{g}}}$$

1B $${{\bf{m}}}^{{\rm{T}}}=\frac{{\sum }_{g=1}^{G}{\sum }_{{i}_{g}=1}^{{{\boldsymbol{I}}}_{g}}{{\bf{m}}}_{{i}_{g}}^{{\rm{T}}}}{{\sum }_{g=1}^{G}{I}_{g}}$$

1C $${{\bf{D}}}_{{\rm{mc}}}={{\bf{X}}}_{\mathrm{log}}-{\bf{1}}{{\bf{m}}}^{{\rm{T}}}$$

where **1** is a column vector of length $\,\sum _{{i}_{g}={1}_{g}}^{{I}_{g}}{N}_{{i}_{g}}$ and matrix **D**_*mc*_ holds the mean-centred data of dimensions equal to **X**.

MFC data has a specific “multiset structure”^25^, *i.e*. single cells are measured per individual. Differences in the number of cells measured for each individual therefore need to be taken into account to base the resulting model equally on all samples. To equally weight each individual in the calculation of the mean, first the individual-specific vector ${{\bf{m}}}_{{i}_{g}}^{{\rm{T}}}$ needs to be calculated, from which the overall mean **m** ^T^ across all individuals can be subsequently calculated. This mean-centring removes the ‘offset’ of the marker expression from the eventual model and puts focus on variability in marker expression among single cells. The multiset structure opens up the possibility for alternative centring approaches. For example, centring each individual using ${{\bf{m}}}_{{i}_{g}}^{{\rm{T}}}$or specific to one of the group ${{\bf{m}}}_{g}^{{\rm{T}}}$, see supplementary.

After centring, eq. 2 scales the centred data to adjust and equalize the relative contributions in expression of each marker within the eventual model.

2A $${{\bf{s}}}^{{\bf{T}}}=\sqrt{\frac{{\sum }_{g=1}^{G}{\sum }_{i=1}^{{I}_{g}}var({{\bf{D}}}_{{\bf{m}}{{\bf{c}}}_{{i}_{g}}})}{{\sum }_{g=1}^{G}{I}_{g}}}$$

2B $${\bf{S}}=\mathrm{diag}{\boldsymbol{(}}{{\bf{S}}}^{{\bf{T}}}{\boldsymbol{)}}\,$$

2C $$\,{{\bf{X}}}_{{\bf{cs}}}={{\bf{D}}}_{{\bf{mc}}}{{\bf{S}}}^{-1}$$

with **X**_cs_ containing the scaled data matrix of size $\,(\sum _{{i}_{g}={1}_{g}}^{{I}_{g}}{N}_{{i}_{g}}\times J)$ and **S** of dimensions (*J* × *J*)a vector where the diagonal element contains the standard deviation of each surface marker across all cells. Note that eq. 2A calculates the mean variance of each centred set, such that the resulting standard deviation is determined by each sample equally.

Scaling removes any differences in the absolute variability in expression between different surface markers that may be caused for example by differences in quantum yield of the used dyes, or in differences in variability in expression between different surface markers, regardless of their bioactivity.

Like for the centring, scaling may also be performed with scaling constants specific to each individual ${{\bf{s}}}_{{i}_{g}}^{{\rm{T}}}$ or specific to one of the groups ${{\bf{s}}}_{g}^{{\bf{T}}}$, see Supplementary.

### Stage 2: Base model

#### PCA (Principal Component Analysis)

Principal Component Analysis (PCA) is the most widely used method for dimension reduction, that reduces the data dimensionality while retaining most of the variability expressed in the originally measured variables^26^.

PCA decomposes the pre-processed data ***X***_cs_ into two matrices^26^ according to eq. 3.

3 $$\,{{\bf{X}}}_{{\rm{cs}}}={{\bf{T}}}_{{\bf{base}}}{{\bf{P}}}_{{\bf{base}}}^{{\bf{T}}}+{\bf{E}}$$

where **T**_**base**_ of size $(\sum _{{i}_{g}={1}_{1}}^{{I}_{G}}{N}_{{i}_{G}}\times \,K)\,\,$contains the PCA scores, *k* = 1, …, *K* indicates the Principal Components (PCs), **P**_**base**_ of size (*K*_base_ × *J*) containing the loadings and **E** being the residuals.

The result of the decomposition consists of single-cell scores

$${{\bf{T}}}_{{\bf{base}}}=\,[\begin{array}{c}{{\bf{T}}}_{{\bf{base}},{1}_{g}}\\ \vdots \\ {{\bf{T}}}_{{\bf{base}},{I}_{G}}\end{array}]$$

that express the cell variability within each individual in ${{\bf{T}}}_{{\bf{base}},{{\rm{1}}}_{g}}\,\,$of dimensions $({N}_{{i}_{g}}\times \,{K}_{{\rm{base}}})$. The contribution of each surface marker to this variability is described in the loadings; both the scores and loadings are expressed in a number of *K*_base_ Principal Components (PCs), where for MFC data $\,{K}_{{\rm{base}}} < J\ll {N}_{{i}_{g}}$. The cell variability that is not contained in this simplified representation is given in the model residual **E**. The loadings of different PCs are mutually orthogonal and therefore constrained as ${{\bf{P}}}_{{\bf{base}}}^{{\rm{T}}}{{\bf{P}}}_{{\bf{base}}}={\bf{I}}$.

When the total number of cells ${N}_{{i}_{g}}$ differs greatly *per* individual, the loadings ${{\bf{P}}}_{{\bf{b}}{\bf{a}}{\bf{s}}{\bf{e}}}^{{\bf{T}}}$ will be highly biased towards those individuals for which most cells have been measured. To avoid this, the standard PCA model in eq. 3 can be slightly adjusted to account for this, see eq. 4.

4A $$\,{{\bf{X}}}_{{\rm{csn}}}=[\begin{array}{c}{{\bf{X}}}_{{1}_{1}}{{\rm{N}}}_{{1}_{1}}^{-1}\,\\ \vdots \\ {{\bf{X}}}_{{I}_{G}}{{\rm{N}}}_{{I}_{G}}^{-1}\end{array}]$$

4B $${{\bf{X}}}_{{\rm{csn}}}={{\bf{T}}}_{{\bf{bas}}{{\bf{e}}}^{\ast }}{{\bf{P}}}_{{\bf{bas}}{{\bf{e}}}^{\ast }}^{{\bf{T}}}+{\bf{E}}$$

4C $${{\bf{T}}}_{{\bf{base}}}={{\bf{X}}}_{{\rm{cs}}}{{\bf{P}}}_{{\bf{bas}}{{\bf{e}}}^{\ast }}$$

The relations between cell variability and the related expression of markers can be combined in a biplot^27^(see Fig. 2).

#### Histogram Construction

The scores ${{\bf{T}}}_{{i}_{g}}\,\,$cannot be quantitatively compared between individuals, as each individual score relates to a single cell within only one individual. In order to describe and then compare the cellular distributions between individuals, we can transform each score submatrix ${{\bf{T}}}_{{{\rm{base}}}_{{i}_{g}}}$ into a histogram matrix ${{\bf{H}}}_{{i}_{g}}$ of size $(\prod _{k=1}^{K}F),\,\,$with *f* = 1, …, *F* indicating the bins within the histograms.

We determine the bin width according to the range of the scores on every PC, according to eq. 5.

5 $${\delta }_{k}=\,\frac{{\bf{percentile}}\,{\bf{99}}.{\bf{95}}({{\bf{t}}}_{k})-{\bf{percentile}}\,{\bf{0}}.{\bf{05}}({{\bf{t}}}_{k})}{F}$$

where **t**_base,*k*_is the vector of length $\sum _{{i}_{g}={1}_{1}}^{{I}_{G}}{N}_{{i}_{G}}\,\,$that contains the base model scores for all cells, for Principal Component *k*_base_. Note that the bin width is determined for all individuals simultaneously, without taking into account the 0.1% extreme values.

Each histogram bin will contain a different number of cells for each histogram ${{\bf{H}}}_{{i}_{g}}$. This is why we normalize each histogram by summing the area under each multidimensional distribution to 1, according to eq. 6.

6 $${{\bf{H}}}_{{i}_{g}}=\,\frac{{{\bf{H}}}_{{i}_{g}}}{{{\rm{N}}}_{{i}_{g}}}$$

As the number of cells that is measured per sample is most often non-informative due to the analytical MFC protocol. Furthermore, the histograms can be plotted together with the marker loadings from the PCA model, resulting in a biplot (Fig. 2). This histogram reveals the cell variability, among which discrete populations can be identified.

#### Histogram Smoothing

Both biological and instrumental variability may cause slight differences in measured marker intensities. This may lead to cells being placed in adjacent bins for different individuals. To reduce the influence of such shifts we apply a Gaussian multidimensional smoothing on every histogram^28^. This smoothing emphasizes the relations of adjacent bins and facilitates their comparability. The binsize *δ*_*k*_ (eq. 5) and the degree of smoothing of this algorithm are highly related parameters, as a histogram with fewer bins requires less smoothing because more cells are captured by the same bin^29^. A number of *F* = 500 bins and smoothing factor 5 provided sufficient resolution in marker expression explained by the PCA scores to be interpretable and to provide sufficient discriminatory power for *K*_base_ = 2. We extended the histogram smoothing algorithm to *K*_base_ > 2 in the Supplementary.

### Stage 3: Top model

The PCA base model in eq. 3 can reveal the relations between many markers with respect to cell variability across many individuals. However, the clinical phenotype of each individual *i*_*g*_ is not used in the Base model. We therefore subject the histograms to supervised, multivariate modelling to find those bins in the histogram that are most distinctive for either control or challenged individuals. We first vectorise the histograms, such that every histogram ${{\bf{H}}}_{{i}_{g}}$ is rearranged into a row vector ${{\bf{h}}}_{{i}_{g}}^{{\rm{T}}}$ of length ${F}^{{K}_{{\rm{base}}}}$. These vectors can then be collected in a second matrix **C** with dimensions $(\sum _{g=1}^{G}{I}_{g}\,\times \,{F}^{{K}_{{\rm{base}}}})$. The systematic differences between the histograms of different groups *g* can be predicted by Orthogonal Partial Least Squares Discriminant Analysis or OPLS-DA^30^. The columns (bins) in matrix **C** with small variance (<10^−6^) are omitted, as these do not contribute much to the top model.

OPLS-DA uses PLS regression^31^ to distinguish multivariate patterns between different groups of samples. Compared to the more conventional PLS-DA algorithm^32^, an Orthogonal Signal Correction (OSC) filter removes all the variation across histograms that is orthogonal and therefore uncorrelated to the response^30, 33^. The latter are encoded in a binary “dummy” vector **y**. The OPLS-DA model is given in eq. 7:

7 $${{\bf{C}}}^{\ast }={{\bf{t}}}_{{\rm{top}}}{{\bf{p}}}_{{\rm{top}}}^{{\bf{T}}}+{{\bf{T}}}_{{\bf{o}}}{{\bf{P}}}_{{\bf{o}}}^{{\bf{T}}}+{{\bf{E}}}_{{\rm{OPLS}}}$$

where **t**_top_ contain the predictive scores of length $\sum _{g=1}^{G}{I}_{g}\,\,$and **p**_top_ of length *F* ^*^ contain the corresponding loadings, matrices **T**_**o**_ and ${{\bf{P}}}_{{\bf{o}}}^{{\bf{T}}}$ are respectively the scores and loadings of the space that is uncorrelated to **y** and are not further considered in this study, like the model residuals in matrix **E**_OPLS_ of size $(\sum _{g=1}^{G}{I}_{g}\,\times \,{F}^{\ast })$.

In addition to the model results above, the OPLS-DA algorithm provides a weight-vector **w**_top_ of length F* that indicates per bin whether it is important for either group in **y**. Scores **t**_top_ indicate how well the challenged individuals can be distinguished from their control counterparts *i*_1_.

### Leukoctye map and Prediction Score

The weight vector can be refolded into an (*F* × *F*) matrix **W**_**top**_, when the components ***K***_**base**_ = 2 in the base model. This corresponds to the original histograms ${{\bf{H}}}_{{{\boldsymbol{i}}}_{{\boldsymbol{g}}}}$, forming a leukocyte map. This map shows which histogram bins, hence which cells, are over- or underrepresented in either group and can be interpreted together with the predictive scores **t**_OPLS_. Each single cell increases the individual score positively or negatively, when it ends up in a positively or negatively weighted bin in the leukocyte map. Low-weight bins do not contribute to the contrast between control and challenged individuals.

### Software

Function for Matlab are available at http://www.ru.nl/science/analyticalchemistry/research/software/.

## Electronic supplementary material

Supplementary
