# Supplementary material for: Novel data analysis method for multicolour flow cytometry links variability of multiple markers on single cells to a clinical phenotype
Source: Sci Rep. 2017 Jul 14;7:5471. doi: 10.1038/s41598-017-05714-1 (PMC5511252; doi:10.1038/s41598-017-05714-1)
Supplement: Supplementary file 1 — Supplementary [file 41598_2017_5714_MOESM1_ESM.pdf]

Novel data analysis method for multicolour flow cytometry links variability of multiple markers on single cells to a clinical phenotype

Gerjen H. Tinnevelt[a,b], Marietta Kokla[a], Bart Hilvering[c], Selma van Staveren[b,c], Rita Folcarelli[a], Luzheng Xue[d], Andries C. Bloem[e], Leo Koenderman[c], Lutgarde M.C. Buydens[a], Jeroen J. Jansen[a]

[a]Radboud University, Institute for Molecules and Materials, (Analytical Chemistry), P.O. Box 9010, 6500 GL Nijmegen, The Netherlands
[b]TI-COAST, Science Park 904, 1098 XH Amsterdam, The Netherlands
[c]Department of Respiratory Medicine, University Medical Center Utrecht, Heidelberglaan 100, 3584CX, Utrecht, The Netherlands
[d]Respiratory Medicine Unit, Nuffield Department of Medicine, University of Oxford, Oxford, OX3 7FZ, United Kingdom
[e]Department of Immunology, University Medical Center, University of Utrecht, 3508GA, Utrecht, The Netherlands

Email: gtinnevelt@science.ru.nl

Index

Figures: 3
Algorithm 8
Overview of pre-processing options visualized with simulated data 12
Data description 17
  Acute myeloid leukaemia data (AML) 17
  Lipopolysaccharide (LPS) challenge 17
  Asthma 18
Four levels 19
Principal Component Analysis (PCA) as top model 20
Multidimensional smoothed histograms; illustrated with the **HIV vaccine trial network data** 24
Analysis of LPS data with ViSNE 27
  Analysis of LPS data with ViSNE 27
  Analysis of LPS data with Citrus 29
References 29

Figures:

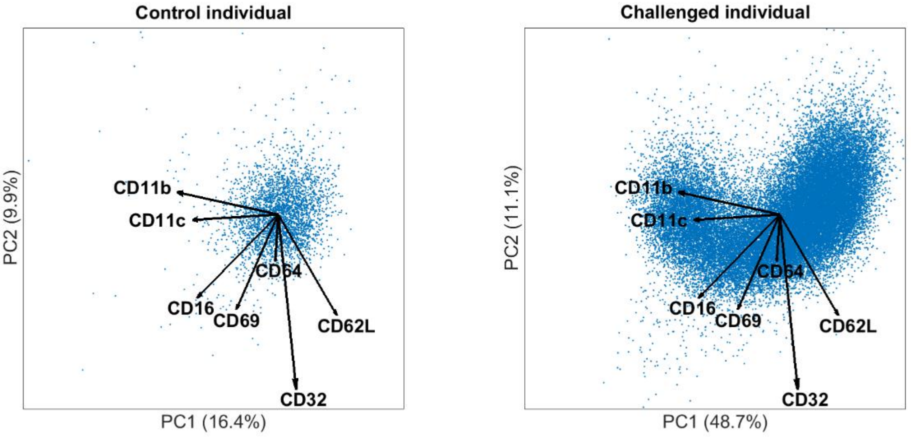

Figure 1: The left panel shows a biplot of the cells of a typical control. The right panel shows a biplot of the cells of a typical LPS-responding individual. Each score is represented by a dot and represents a single cell. The model loadings are represented by vectors and indicate how each surface marker contributes to the cell variability in a specific direction within the model, as described in the paragraph Base model.

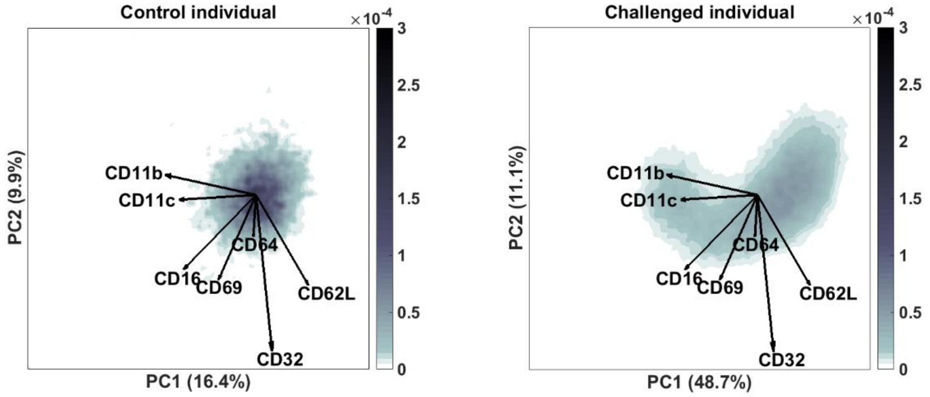

Figure 2: Smoothed histograms of the biplots shown in Figure 2. The right panel shows a histogram of a typical LPS-responding individual. The darker bin, the more cells are likely present in that location based on the same PCA as Figure 1. The same loadings are plotted on top as vectors and indicate how each surface marker contributes to the cell variability in a specific direction within the model.

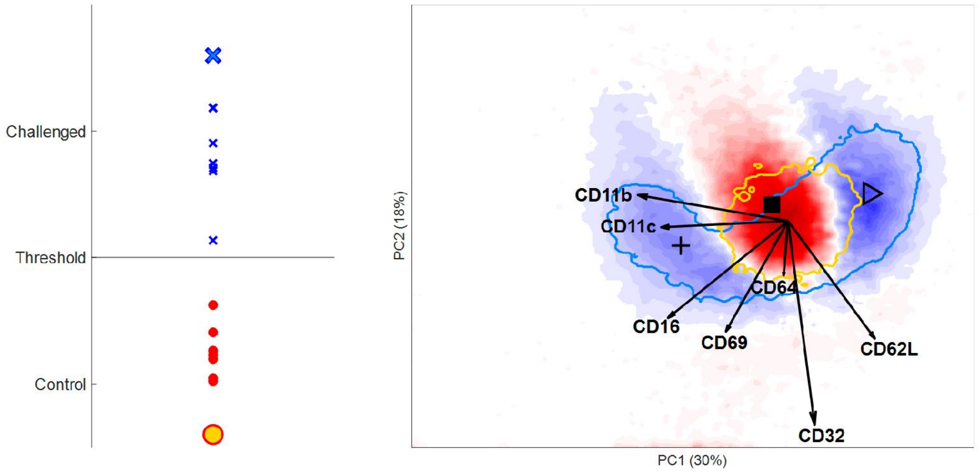

Figure 3: DAMACY model of LPS data. The left panel shows the prediction score of the OPLS-DA model of controls as red rounds and LPS-responding individuals as blue crosses. The right panel shows negative weights as red and positive weights as blue. The blue contour depicts where most cells of the enlarged cross are. The red contour depicts where most cells of the enlarged round are. The loadings of the Base model are plotted on top as black vectors and indicate how each surface marker contributes to the cell variability in a specific direction within the model.

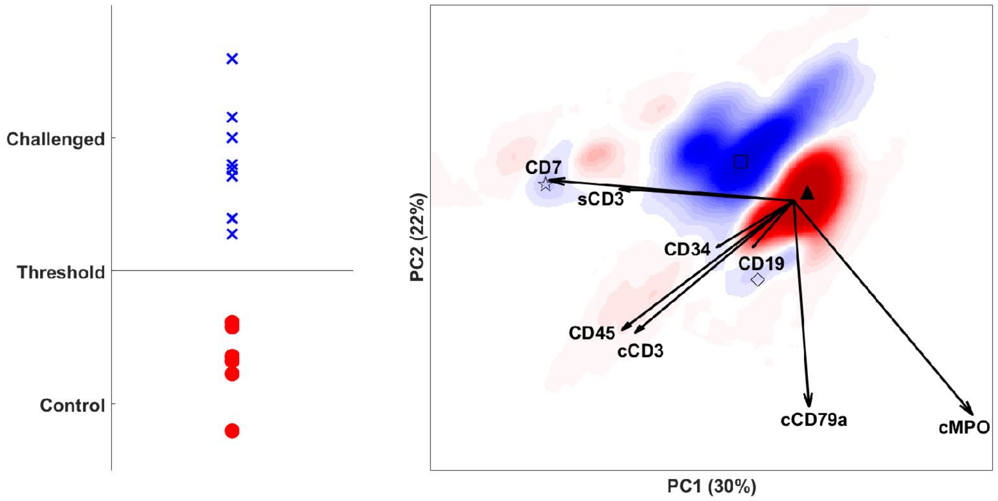

Figure 4: DAMACY model of AML data. The left panel shows the average prediction score of controls (red rounds) and AML patients (blue crosses). The right panel shows negative weights as red and positive weights as blue. The loadings of the Base model are plotted on top as black vectors and indicate how each marker contributes to the cell variability in a specific direction

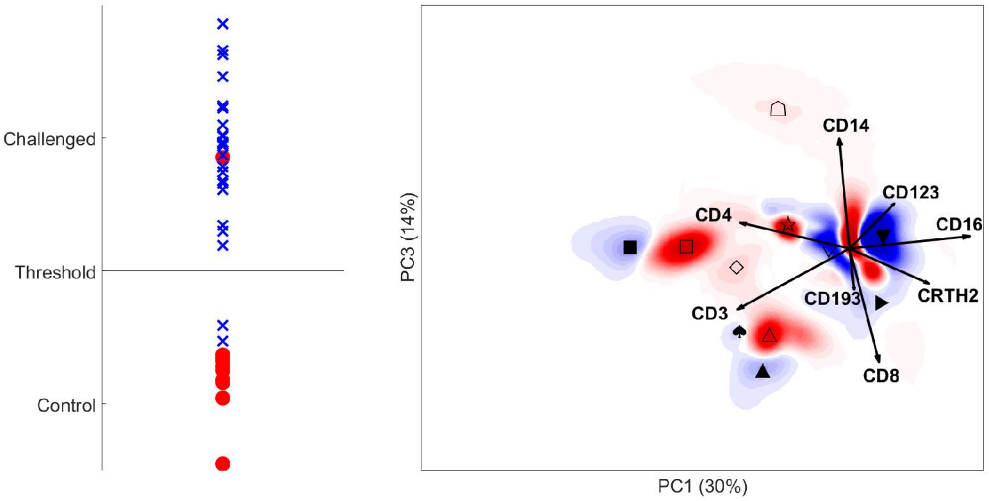

Figure 5: DAMACY model of asthma data. The left panel shows the average prediction score of the OPLS-DA model of controls as red rounds and asthma individuals as blue crosses. The right panel shows negative weights as red and positive weights as blue. The loadings of the Base model are plotted on top as black vectors and indicate how each surface marker contributes to the cell variability in a specific direction within the model. The symbols indicate the centroid of that coloured area.

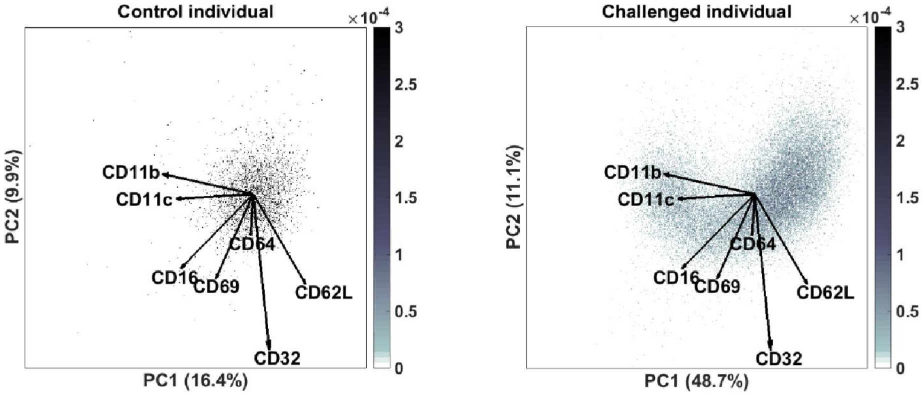

Figure s1: Unsmoothed histograms of the biplots shown in Figure 1. The right panel shows a histogram of a typical LPS-responding individual. The darker bin, the more cells are present in that location based on the same PCA as Figure 1. The same loadings are plotted on top as vectors and indicate how each surface marker contributes to the cell variability in a specific direction within the model.

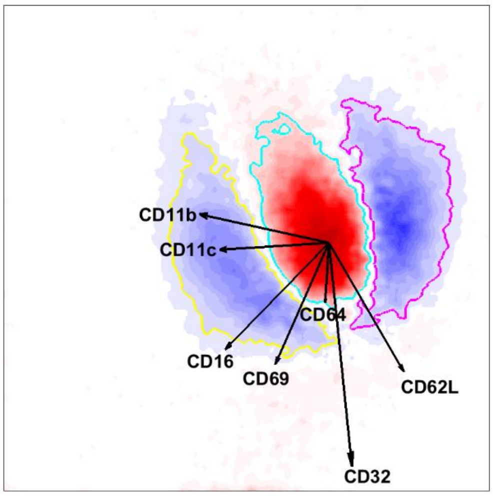

Figure s2: DAMACY immunological cell map, which is the same as in the right panel of Figure 4. Negative weights are depicted as red and positive weights as blue. Now three regions are highlighted based on the intensity weights. The original cells that lie inside these contours can be gated.

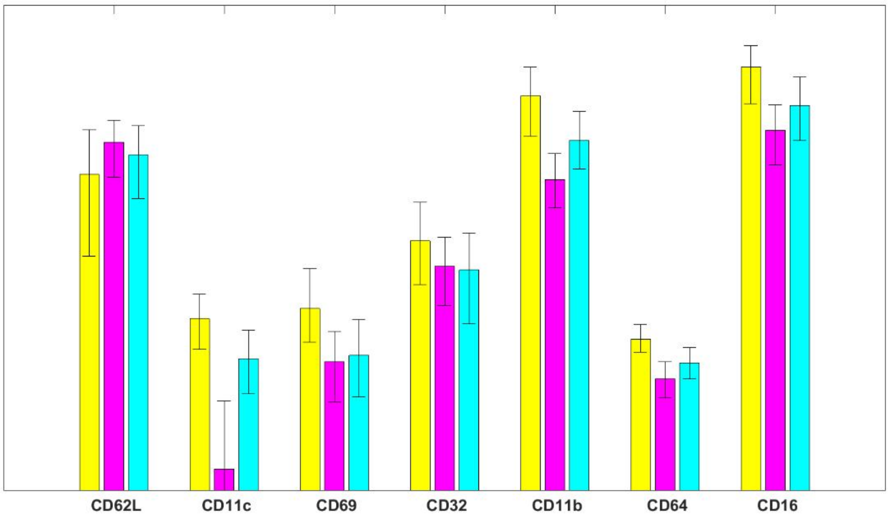

Figure s3: Shows the original median logarithmic fluorescence intensity of each marker of each gate depicted with the same colors as in Figure s2.

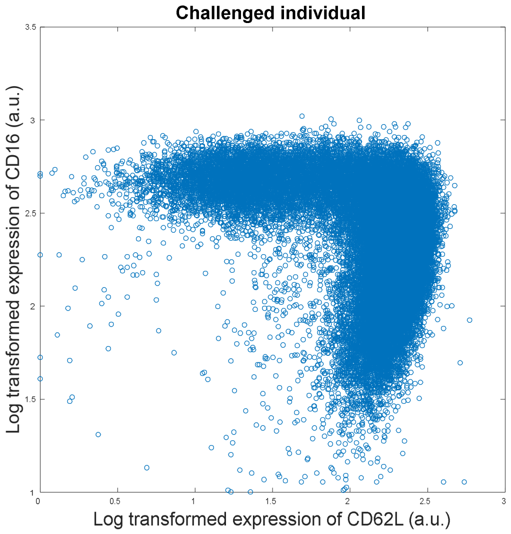

Figure s4: Expression of CD62L and CD16 of a challenged individual.

Figure s5: Schematic overview of the DAMACY algortihm

Algorithm

Below you find the complete algorithm of Discriminant Analysis of Multi Aspect flow Cytometry data (DAMACY):

1) Import the data and arrange it as matrix $\mathbf{X} = \begin{bmatrix} \mathbf{X}_{1_1} \\ \vdots \\ \mathbf{X}_{I_G} \end{bmatrix}$ of size $\left(\sum_{i_g=1_1}^{I_G} N_{i_G} \times J\right)$ for analysis by DAMACY, where N_{i_g} is the number of cells per individual $i_g = 1_1, \ldots I_1, \ldots 1_G, \ldots I_G$; $g = 1, \ldots\ldots G$ indicates the pre-defined groups of individuals with $g = 1$ representing the control group and $g = 2$ the patient/challenged group, with $j = 1, \ldots, J$ indicates the surface markers; The data of each individual can analogously be denoted as $\mathbf{X}_{i_g} = \begin{bmatrix} \mathbf{x}_{1_{i_g}}^T \\ \vdots \\ \mathbf{x}_{N_{i_g}}^T \end{bmatrix}$ of size $\left(N_{i_g} \times J\right)$ and the data of each class as $\mathbf{X}_g = \begin{bmatrix} \mathbf{X}_{1_g} \\ \vdots \\ \mathbf{X}_{I_g} \end{bmatrix}$ of size $\left(\sum_{i_g=1_g}^{I_g} N_{i_g} \times J\right)$.
2) Set all negative values to 1 and log-transform the data: $\mathbf{X}_{\log} = \mathbf{log}_{10}(\mathbf{X})$
3) Separate the data into test and training set using leave one of each group out validation for small datasets or use 7 cross-validation for larger datasets (>50 individuals per class) or use a predetermined training and test set like the FLOWCAP II challenge.
4) Separate the training data in a training set and a calibration set using leave one of each group out validation for small datasets or use 7 cross-validation for larger datasets (>50 individuals per group).
5) Mean centre the data using equation 1 or supplementary equation s1, s2. Note: only use training data to calculate mean of multiple individuals.
6) Scale the data using equation 2 or supplementary equation s3,s4. Note: only use training data to calculate standard deviation of multiple individuals.
7) Perform simultaneous component analysis (SCA) on training data using equation 4.
8) Project the test data onto the SCA model using equation 4C.
9) Define binsize using equation 5.
10) Create histograms $\mathbf{H}$ of size $\left(\sum_{g=1}^{G} I_g \times \prod_{k_{base}=1}^{K_{base}} F\right)$, with $k_{base} = 1, \ldots, K_{base}$ indicating the components used in the base model, $f = 1, \ldots, F$ indicating the bins within the histograms, by counting the number of cells per bin per individual and normalize it using equation 6.
11) Smooth the histogram using supplementary equation s5, with $\mathbf{I}$ being the identity matrix, for matrix $\mathbf{D}_1$ holds that $\mathbf{D}_1\mathbf{H} = \Delta\mathbf{H}$ and for matrix $\mathbf{D}_2$ holds that $\mathbf{D}_2\mathbf{H} = \Delta^2\mathbf{H}$, λ is the smoothing factor, this equation results in smoothed histogram $\hat{\mathrm{H}}_{i_g}$ of size $\left(\prod_{k_{base}=1}^{K_{base}} F\right)$

12) Refold the smoothed histograms Ĥ into matrix **C** with dimensions$\left(\sum_{g=1}^{G} I_g \times F^{K_{\text{base}}}\right)$
13) Remove the columns (bins) in matrix **C** with small variance ($< 10^{-6}$), resulting in matrix $\mathbf{C}^*$ with dimensions $\left(\sum_{g=1}^{G} I_g \times F^*\right)$, with F^* being the bins with sufficient variance.
14) Perform Orthogonal Partial Least Squares – Discriminant Analysis (OPLS-DA) on train data with equation 7
15) Predict the test data with $\hat{y} = (\mathbf{C}^*_{\text{test}} - \mathbf{C}^*_{\text{test}}\mathbf{W_o}\mathbf{P_o^T})\mathbf{w}_{\text{top}}\mathbf{p}_{\text{top}}^{\mathbf{T}}\mathbf{q}$
16) Go back to step 4 until you have a prediction for every individual in the training set for every model based on the different settings of parameters such as way of pre-processing, principle components used in the base model, number of bins, smoothing factor and number of orthogonal latent variables in the OPLS-DA top model.
17) Based on the prediction of calibration set select the optimal parameters.
18) Go back to step 3 until you have a prediction for every individual using the optimal parameters.
19) Perform OPLS-DA on complete data with equation 7
20) Refold weight vector $\mathbf{w}_{\text{top}}$ into an $(F \times F)$ **matrix** $\mathbf{W_{top}}$, when the components $K_{\text{base}} = 2$ in the base model.
21) Plot matrix $\mathbf{W_{top}}$ with red intensity for negative weights and blue intensity for positive weights.

Equations used in the algorithm:

(1) **A**

$$\mathbf{m}_{i_g}^{\mathrm{T}} = \frac{\sum_{n=1}^{N_{i_g}} \mathbf{X}_{\log_{i_g}}}{N_{i_g}}$$

B

$$\mathbf{m}^{\mathrm{T}} = \frac{\sum_{g=1}^{G} \sum_{i_g=1}^{I_g} \mathbf{m}_{i_g}^{\mathrm{T}}}{\sum_{g=1}^{G} I_g}$$

C

$$\mathbf{D}_{\mathrm{mc}} = \mathbf{X}_{\log} - \mathbf{1}\mathbf{m}^{\mathrm{T}}$$

(s1) **A**

$$\mathbf{m}_{i_1}^{\mathrm{T}} = \frac{\sum_{n=1}^{N_{i_1}} \mathbf{X}_{\log_{i_1}}}{N_{i_1}}$$

B

$$\mathbf{m}_1^{\mathrm{T}} = \frac{\sum_{i=1}^{I_1} \mathbf{m}_{i_1}^{\mathrm{T}}}{I_1}$$

C

$$\mathbf{D}_{\mathrm{mc}} = \mathbf{X}_{\log} - \mathbf{1}\mathbf{m}_1^{\mathrm{T}}$$

(s2) **A**

$$\mathbf{m}_{i_g}^{\mathrm{T}} = \frac{\sum_{n=1}^{N_{i_g}} \mathbf{X}_{\log_{i_g}}}{N_{i_g}}$$

B

$$\mathbf{D}_{\mathrm{mc}_{i_g}} = \mathbf{X}_{\log_{i_g}} - \mathbf{1}_{i_g} \mathbf{m}_{i_g}^{\mathrm{T}}$$

(2) **A**

$$\mathbf{s}^{\mathbf{T}} = \sqrt{\frac{\sum_{g=1}^{G} \sum_{i=1}^{I_g} var\left(\mathbf{D}_{\mathbf{mc}_{i_g}}\right)}{\sum_{g=1}^{G} I_g}}$$

B

$$\mathbf{S} = \mathbf{diag}\left(\mathbf{s}^{\mathbf{T}}\right)$$

C

$$\mathbf{X}_{\mathbf{cs}} = \mathbf{D}_{\mathbf{mc}} \mathbf{S}^{-\mathbf{1}}$$

(s3) **A**

$$\mathbf{s}_1^{\mathbf{T}} = \sqrt{\frac{\sum_{i=1}^{I_1} var\left(\mathbf{D}_{\mathbf{mc}_{i_1}}\right)}{I_1}}$$

B

$$\mathbf{S}_1 = \mathbf{diag}\left(\mathbf{s}_1^{\mathbf{T}}\right)$$

C

$$\mathbf{X}_{\mathbf{cs}} = \mathbf{D}_{\mathbf{mc}} \mathbf{S}_1^{-\mathbf{1}}$$

(s4) **A**

$$\mathbf{S}_{\boldsymbol{i_g}} = \mathbf{diag}\left(\mathbf{s}_{i_g}^{\mathbf{T}}\right)$$

B

$$\mathbf{X}_{\mathbf{cs}_{\boldsymbol{i_g}}} = \mathbf{D}_{\mathbf{mc}_{i_g}} \mathbf{S}_{i_g}^{-\mathbf{1}}$$

(4) **A**

$$\mathbf{X}_{\mathbf{csn}} = \begin{bmatrix} \mathbf{X}_{\mathbf{1_1}} \mathbf{N}_{\mathbf{1_1}}^{-\mathbf{1}} \\ \vdots \\ \mathbf{X}_{\boldsymbol{I_G}} \mathbf{N}_{\boldsymbol{I_G}}^{-\mathbf{1}} \end{bmatrix}$$

B $$\mathbf{X_{csn}} = \mathbf{T_{base*}P_{base*}^{T}} + \mathbf{E}$$

C $$\mathbf{T_{base}} = \mathbf{X_{cs}P_{base*}}$$

(5) $$\delta_k = \frac{\mathbf{percentile99.95(t}_k) - \mathbf{percentile0.0\ 5(t}_k)}{F}$$

(6) $$\mathbf{H}_{i_g} = \frac{\mathbf{H}_{i_g}}{\mathrm{N}_{i_g}}$$

(s5) $$(\mathbf{I} + \mathbf{2}\ \lambda \mathbf{D_1^T D_1} + \lambda^2 \mathbf{D_2^T D_2})\ \hat{\mathrm{H}}_{i_g} = \mathbf{H}_{i_g}$$

(7) $$\mathbf{C}^* = \mathbf{t}_{\mathrm{top}}\mathbf{p}_{\mathrm{top}}^{\mathbf{T}} + \mathbf{T_o P_o^T} + \mathbf{E}_{\mathrm{OPLS}}$$

Overview of pre-processing options visualized with simulated data

Like discussed in the main paper, multivariate analysis is used to reduce or remove artefacts to enhance the biologically relevant information. Centring is used to remove shifts from the data and scaling is used to remove the differences in scales of marker expression; each marker becomes equally important. These two pre-processing steps can be used in three different ways due to the unique "multiset structure" of flow cytometry data, *i.e.* multiple single cells are measured for each individual. The three ways are to centre/scale using the mean/standard deviation of cells per individual, of all control individuals or of all individuals.

These different pre-processing strategies were tested on simulated data. These simulated data consisted of two normal distributions, mimicking a cell population. One 'control' distribution was drawn from a normal distributions with true mean μ = 4,0 and true standard deviation σ = 0.5, 2 and the other 'challenged' distribution from a normal distribution with true mean μ = 4,4 and true standard deviation σ = 2, 0.5, seen in Figure s6. This figure shows that the challenged population has a different shape and is shifted compared to the control population.

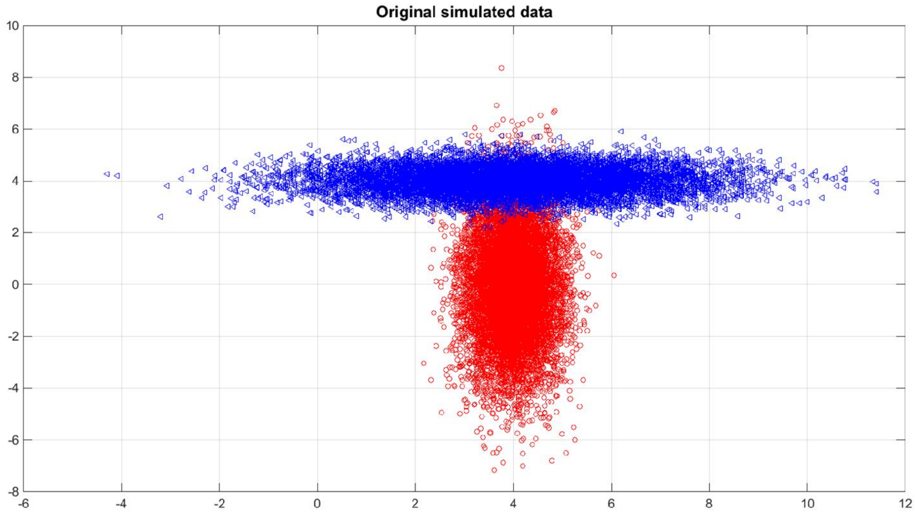

Figure s6: Shows the simulated cells in red for 'control' population and in blue for the 'challenged' population

Multicolour flow cytometry (MFC) data can be arranged into matrix $\mathbf{X} = \begin{bmatrix} \mathbf{X}_{1_1} \\ \vdots \\ \mathbf{X}_{I_G} \end{bmatrix}$ of size $\left(\sum_{i_g=1_1}^{I_G} N_{i_G} \times J\right)$ for data analysis, where N_{i_g} is the number of cells per individual $i_g = 1_1, \dots I_1, \dots 1_G, \dots I_G$; $g = 1, \dots .. G$ indicates the pre-defined groups of individuals with $g = 1$ representing the control group and $g = 2$ the patient/challenged group, with $j =$

$1, \dots, J$ indicates the surface markers. The data of each individual can analogously be denoted as $\mathbf{X}_{i_g} = \begin{bmatrix} \mathbf{x}_{1_{i_g}}^T \\ \vdots \\ \mathbf{x}_{N_{i_g}}^T \end{bmatrix}$ of size $\left(N_{i_g} \times J\right)$ and the data of each class as $\mathbf{X}_g = \begin{bmatrix} \mathbf{X}_{1_g} \\ \vdots \\ \mathbf{X}_{I_g} \end{bmatrix}$ of size $\left(\sum_{i_g=1_g}^{I_g} N_{i_g} \times J\right)$. Prior to pre-processing all negative values are set to 1 and data is log transformed $\mathbf{X}$ to $\mathbf{X}_{\log} = \mathbf{log}_{10}(\mathbf{X})$.

The first three figures show the centring without any scaling performed and visualize the following:

Centring based on the control individuals is given by equation s1:

(s1) **A**

$$\mathbf{m}_{i_1}^{\mathrm{T}} = \frac{\sum_{n=1}^{N_{i_1}} \mathbf{X}_{\log_{i_1}}}{N_{i_1}}$$

B

$$\mathbf{m}_1^{\mathrm{T}} = \frac{\sum_{i=1}^{I_1} \mathbf{m}_{i_1}^{\mathrm{T}}}{I_1}$$

C $$\mathbf{D}_{\mathrm{mc}} = \mathbf{X}_{\log} - \mathbf{1}\mathbf{m}_1^{\mathrm{T}}$$

With $\mathbf{m}_{i_g}^{\mathrm{T}}$ being the average surface marker expression of one individual. The overall mean $\mathbf{m}_1^{\mathrm{T}}$ is the average surface marker expression based on all controls. **1** is a column vector of length $\sum_{g=1}^{G} \sum_{i_g=1}^{I_g} N_{i_g}$ and matrix $\mathbf{D}_{mc}$ holds the mean-centred data of dimensions equal to **X**. Note that only the cells of the controls are taken into account, denoted with $\mathbf{X}_{\log_{i_1}}$. Centring based on the control individuals will remove the shift of the control cells and will emphasize the difference between control (new mean $m_{\mathrm{control}} = 0{,}0$) and challenged (new mean $m_{\mathrm{challenged}} = 0{,}2$).

Centring based on all the data from both the groups is given by equation 1:

(1) **A**

$$\mathbf{m}_{i_g}^{\mathrm{T}} = \frac{\sum_{n=1}^{N_{i_g}} \mathbf{X}_{\log_{i_g}}}{N_{i_g}}$$

B

$$\mathbf{m}^{\mathrm{T}} = \frac{\sum_{g=1}^{G}\sum_{i_g=1}^{I_g}\mathbf{m}_{i_g}^{\mathrm{T}}}{\sum_{g=1}^{G} I_g}$$

C $$\mathbf{D}_{\mathrm{mc}} = \mathbf{X}_{\log} - \mathbf{1}\mathbf{m}^{\mathrm{T}}$$

The overall mean $\mathbf{m}^{\mathbf{T}}$ is the average marker expression based on all individuals. Centring based on all the data will put both groups around the origin ($m_{control}$ = 0,-2; $m_{challenged}$ = 0,2). Note that here the complete dataset is taken, denoted with $\mathbf{X}_{\log_{i_g}}$ in equation 1A.

Finally, centring per individual is given by equation s2:

(s2) **A** $$\mathbf{m}_{i_g}^{\mathrm{T}} = \frac{\sum_{n=1}^{N_{i_g}}\mathbf{X}_{\log_{i_g}}}{N_{i_g}}$$

B $$\mathbf{D}_{\mathrm{mc}_{i_g}} = \mathbf{X}_{\log_{i_g}} - \mathbf{1}_{i_g}\mathbf{m}_{i_g}^{\mathrm{T}}$$

Where $\mathbf{1}_{i_g}$ is a column vector of length N_{i_g}. Finally, centring per individual will remove the individual shift of both groups ($m_{control}$ = $m_{challenged}$ = 0,0). Note that the mean centring is done per individual, resulting in an individual mean-centred data matrix $\mathbf{D}_{\mathrm{mc}_{i_g}}$, seen in equation s2B.

Scaling based on the control population is given in equation s3:

(s3) **A** $$\mathbf{s}_1^{\mathbf{T}} = \sqrt{\frac{\sum_{i=1}^{I_1} var\left(\mathbf{D}_{\mathbf{mc}_{i_1}}\right)}{I_1}}$$

B $$\mathbf{S}_1 = \mathrm{diag}(\mathbf{s}_1^{\mathbf{T}})$$

C $$\mathbf{X}_{\mathbf{cs}} = \mathbf{D}_{\mathbf{mc}}\mathbf{S}_1^{-\mathbf{1}}$$

with $\mathbf{X}_{\mathrm{cs}}$ containing the scaled data matrix of size $\left(\sum_{g=1}^{G}\sum_{i_g=1}^{I_g} N_{i_g} \times J\right)$ and $\mathbf{S}_1$ of dimensions $(J \times J)$ vector where the diagonal element contains the standard deviation $\mathbf{s}_1^{\mathbf{T}}$ of each surface marker based on all cells of control individuals. Scaling based on the control population (σ = 0.5, 2) will remove the shape of the control (new standard deviation $s_{control}$ = 1, 1) and emphasize

the shape of the challenged population (new standard deviation $s_{challenged} = 4, 0.25$). Note that the standard deviation is based on only the control individuals $\mathbf{D}_{\text{mc}_{i_1}}$.

Scaling based on the complete data is given in equation 2:

(2) **A**
$$\mathbf{s}^{\mathbf{T}} = \sqrt{\frac{\sum_{g=1}^{G}\sum_{i=1}^{I_g} var\left(\mathbf{D}_{\mathbf{mc}_{i_g}}\right)}{\sum_{g=1}^{G} I_g}}$$

B $$\mathbf{S} = \mathbf{diag}(\mathbf{s}^{\mathbf{T}})$$

C $$\mathbf{X}_{\mathbf{cs}} = \mathbf{D}_{\mathbf{mc}}\mathbf{S}^{-\mathbf{1}}$$

Where $\mathbf{S}$ of dimensions $(J \times J)$ vector where the diagonal element contains the standard deviation of each surface marker $\mathbf{s}^{\mathbf{T}}$ based on all cells all individuals. Scaling based on the complete data will keep the same difference in shape between control ($s_{control} = 0.34, 1.37$) and challenged ($s_{challenged} = 1.37, 0.34$), but the variables are now equally important ($s = 1,1$).

Scaling based on the complete data is given in equation s4:

(s4) **A** $$\mathbf{S}_{i_g} = \mathbf{diag}\left(\mathbf{s}_{i_g}^{\mathbf{T}}\right)$$

B $$\mathbf{X}_{\mathbf{cs}_{i_g}} = \mathbf{D}_{\mathbf{mc}_{i_g}}\mathbf{S}_{i_g}^{-\mathbf{1}}$$

Where $\mathbf{S}_{i_g}$ of dimensions $(J \times J)$ vector where the diagonal element contains the standard deviation of each surface marker $\mathbf{s}_{i_g}^{\mathbf{T}}$ based on all cells of one individuals. Scaling per individual will remove the shape of both groups ($s_{control} = s_{challenged} = 1, 1$).

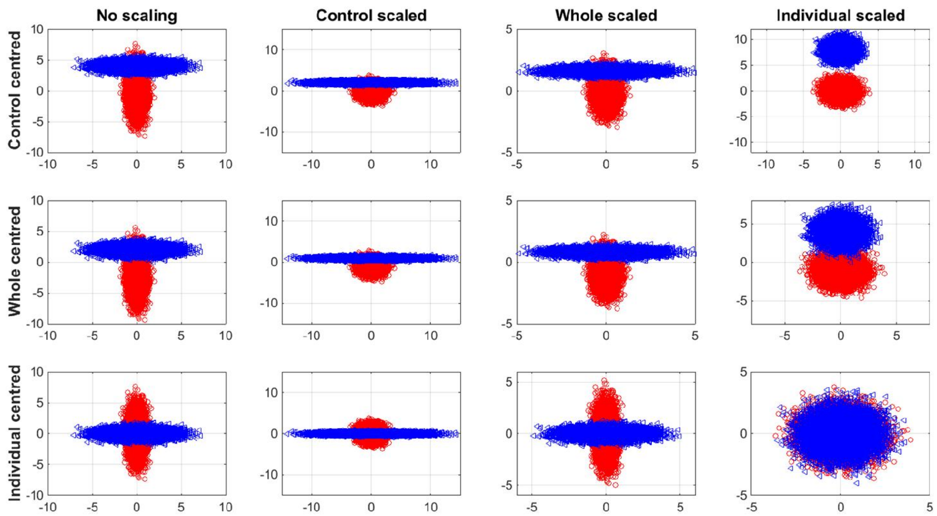

Figure s7: Shows the resulting pre-processed simulated data for each pre-processing combination. The red rounds are cells belonging to the 'control' population and the blue triangles are cells belonging to the 'challenged' cell population. The columns contain the different scaling option going from no scaling, scaling based on controls, scaling based on complete data to individual scaling. The rows show the different centring options from centring based on controls, centring based on complete data to centring per individual.

This simulation in Figure s7 shows that based on the way of pre-processing, the resulting data structure can be very different. One of the interesting options is centring and scaling based on only the control individuals, which emphasizes the difference between control and challenged cell populations. Another interesting way is the individual centring and scaling wich leads to two identical cell populations, this is can be used if you are only interested in the representation of this cell population and not in shifts of the populations.

We found that in the case of asthma and in the lipopolysaccharide challenge data, the best prediction was achieved when we used individual centring and scaling based on all the individuals. In this way you keep the difference in expression of surface markers, but shifts in the data are removed. In the Acute Myeloid Leukemia (AML) data large shifts were observed, which were expected since the leukemic cells can express aberrant markers which normal muyeloid cells do not express. This is why centring and scaling based on all the cells yielded the best results.

Data description

Subjects with asthma and normal subjects participating in the LPS challenge study gave peripheral blood. All data were obtained by using standardized protocols. All studies and sample collection were approved by the medical ethics committees of University Medical Centers Utrecht (asthma patients/UMCU) and Radboud University Medical Center (LPS challenge/RadboudUMC), the Netherlands. These participants gave their written informed consent. AML data were from bone marrow and were extracted from Anonymized flow cytometry list mode data sets (fcs files). These data sets were originally acquired as part of the UMCUtrecht institutional work up scheme for evaluation of leukaemia.

Acute myeloid leukaemia data (AML)

Bone marrow samples were analysed for the presence of AML by MFC for diagnostical purposes using the acute leukemia orientation tube (ALOT) as described by the EuroFlow consortium.[1] The ALOT tube is a single 8-color-tube designed for the identification of an expanded population of immature blast like cells, including AML blasts. The 8-color panel consists of detection of CD3 (cytoplasmic and surface), MPO (cytoplasmic), CD79a (cytoplasmic) and the surface expression of CD19, CD34 CD45 and CD7 (). The uncompensated list mode fcs data files of bone marrow samples (100.000 stained cells) of 9 AML patients and 6 individuals where no haematological malignancy could be detected in the BM, were used in this study. The flowcytometric analyses were performed on a FACSCanto II (Becton Dickinson).

Lipopolysaccharide (LPS) challenge

In the LPS challenge dataset there are 16 individuals: 8 healthy controls and 8 that have undertaken the LPS challenge. Flow Cytometry measurements were performed during an endotoxin trial (NCT01374711; www.clinicaltrials.gov). Details regarding the Flow Cytometry experiments that provided the data to illustrate the method are described in the Online Supplement I; source data can be accessed through www.flowrepository.org ID: FR-FCM-ZZEE. This dataset has been extensively analysed in two earlier publications.

Asthma

The asthma dataset contains 24 asthma patients and 10 healthy controls aged 18-75 were recruited at the respiratory outpatient clinics of the Churchill Oxford University Hospital, UK. The study received ethical approval, and written informed consent was obtained. After inclusion patients filled out symptom questionnaires, sputum induction was performed, blood was taken, and patients underwent FeNO measurement and lung functional testing. All patients were receiving appropriate asthma treatment at the time of blood withdrawal. Blood cells were stained with a panel of 8 antibodies including CD3, CD4, CD8, CD14, CD16, CRTH2 (CD294), CD123 and CD193. After staining, red blood cells were lysed using a FACS Lysing solution (Becton Dickinson). Cells were measured on a LSR Fortessa flow cytometer (Becton Dickinson).

Four levels

The leukocyte map, together with the prediction scores quantitatively connect all four information levels within MFC data. The associations between the co-expressions of different markers can be evaluated from the Base model loadings $\mathbf{P}_{\text{base}}$ (level 1). The cells for which these related markers are most prominently over or underrepresented can be observed from the interpretation of $\mathbf{W}_{\text{top}}$. From the latter, co-varying bins in the histograms $\mathbf{H}_{i_g}$ can aggregate in regions, which may correspond to cell populations (level 2). The degree to which an individual responds to the entire contrast $\mathbf{W}_{\text{top}}$ can be found by superimposing the histogram $\mathbf{H}_{i_g}$ (Level 3). Whether the haematological map in fact represents a specific clinical phenotype can be determined from the classification (*i.e.* sensitivity and selectivity) of these scores (Level 4).

Principal Component Analysis (PCA) as top model

Instead of a classification, the Top model can also be an ordination to explore the heterogeneity in a more unsupervised fashion; the model in equation 7 can then be replaced by a PCA model that provides descriptive rather than predictive scores $\mathbf{T}_{\mathrm{top,PCA}}$. The loadings of these components are then however less intuitively interpretable, because they are not directly related to a classification between groups. However, it is possible to define a line in the PC space and to project the loadings onto this line, in order to see the difference between control and case clusters or completely different observed clusters. This is quite tedious work as you have to subjectively define the line and for every line you get a different map.

Quantitative analysis of the variability between the histograms in a Top PCA model shows that the major variability across the histograms occurs between those of controls and of challenged individuals, as seen in Figure s8. Secondly, the variability in surface marker expression across control individuals is larger than that between case individuals. Thirdly, three control individuals deviate strongly from the other five control individuals as well as from the challenged individuals; a similar yet less-pronounced heterogeneity is observed between two clusters of challenged individuals.

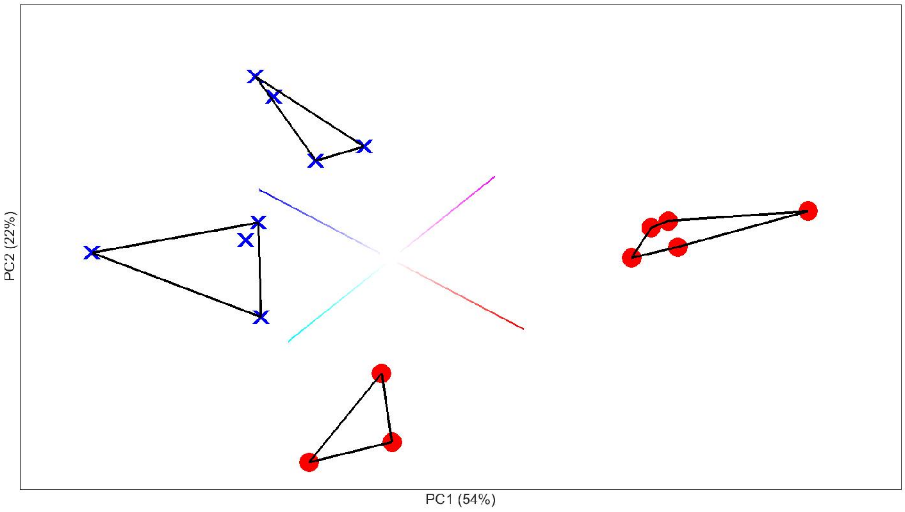

Figure s8: Top PCA score plot. Each red round represents a control and each blue cross a LPS-responding individual. Four cluster can be found and are shown with a black convex hull. The red-blue line is the direction from mean of the two control clusters, depicted in red, to the mean of the two LPS-responding clusters, depicted in blue. The magenta-cyan line is in the direction from mean of the top control and top LPS-responding clusters, depicted in magenta, to the mean of the bottom control and bottom LPS-responding clusters, depicted in cyan.

The Top PCA however lacks in two aspects: Firstly, the separation is based on maximum variability between all individuals; the distinction between challenged individuals and controls can only be observed if the immune response is considerably larger than the variability in surface marker expression between individuals. Secondly, if the Top PCA shows a distinction between scores of challenged individuals and controls, the response can only be characterized through a linear combination between two Principal Components, as seen in Figure s9 and s10. Figure s11 shows the Top PCA model for the asthma dataset and shows some grouping between the individuals but no distinction between the asthma and controls. Figure s12 shows a great distinction between the top PCA scores of controls and AML patients, however, no clear objective line can be defined to separate all the controls from the all AML patients.

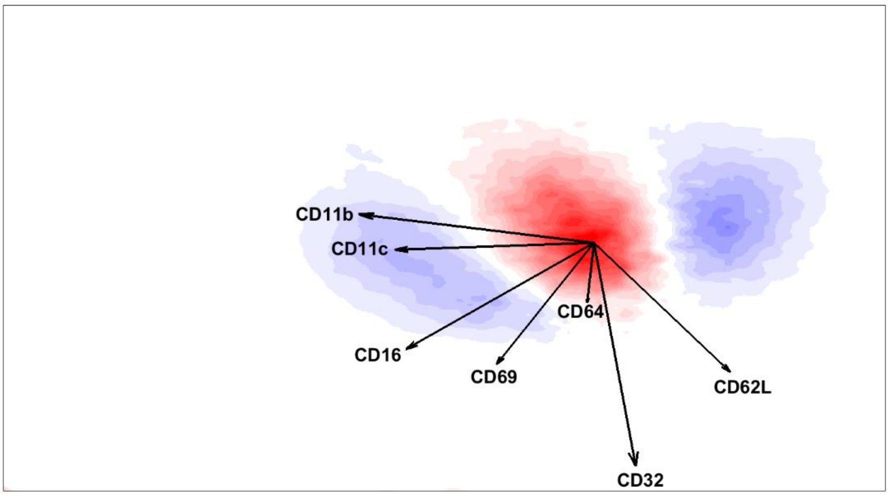

Figure s9: Shows the linear combination of loadings from the top PCA model seen in Figure s8. This is based on the red-blue line, observed in Figure s8, in the direction from mean of the two control clusters, depicted in red, to the mean of the two LPS-responding clusters, depicted in blue. The loadings of the Base model are plotted on top as black vectors and indicate how each surface marker contributes to the cell variability in a specific direction within the model.

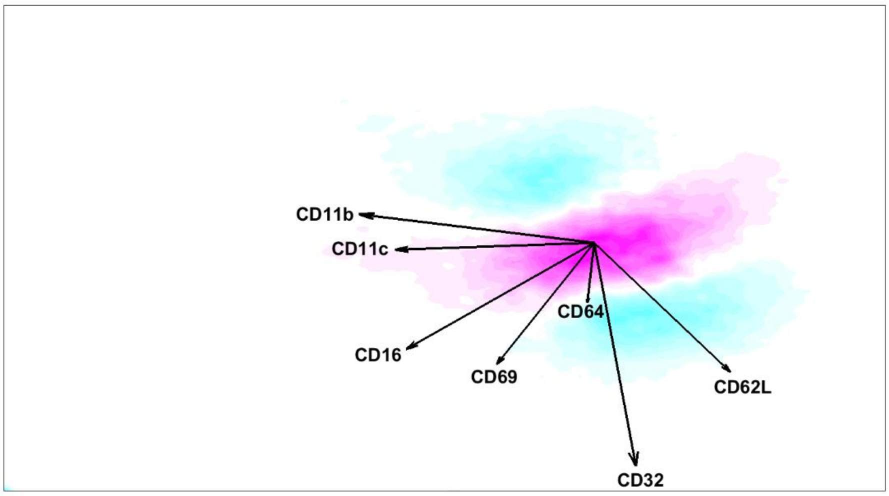

Figure s10: Shows the linear combination of loadings from the top PCA model seen in Figure s8. This is based on the magenta-cyan line, observed in Figure s8, in the direction from mean of the top control and top LPS-responding clusters, depicted in magenta, to the mean of the bottom control and bottom LPS-responding clusters, depicted in cyan. The loadings of the Base model are plotted on top as black vectors and indicate how each surface marker contributes to the cell variability in a specific direction within the model.

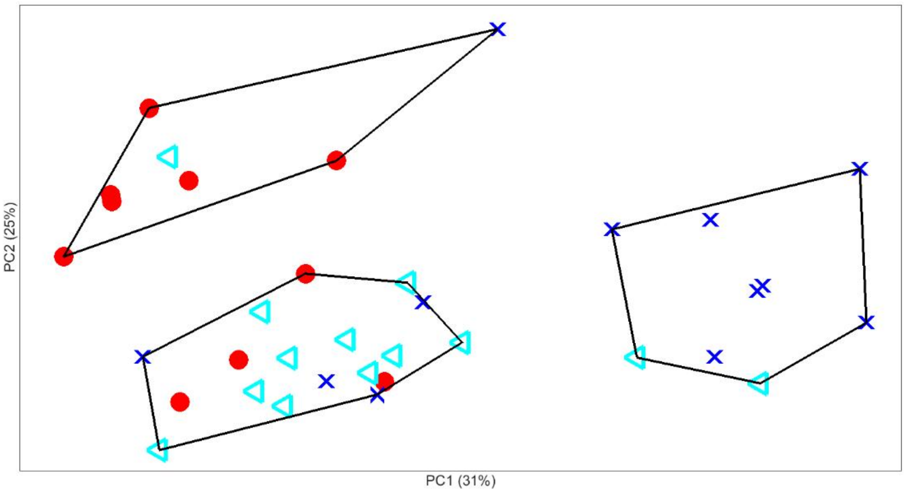

Figure s11: Top PCA score plot. Each red round represents a control, each cyan triangle represents a mild asthma patient and each blue cross a severe asthma patient. Three clusters can be found and are shown with a black convex hull.

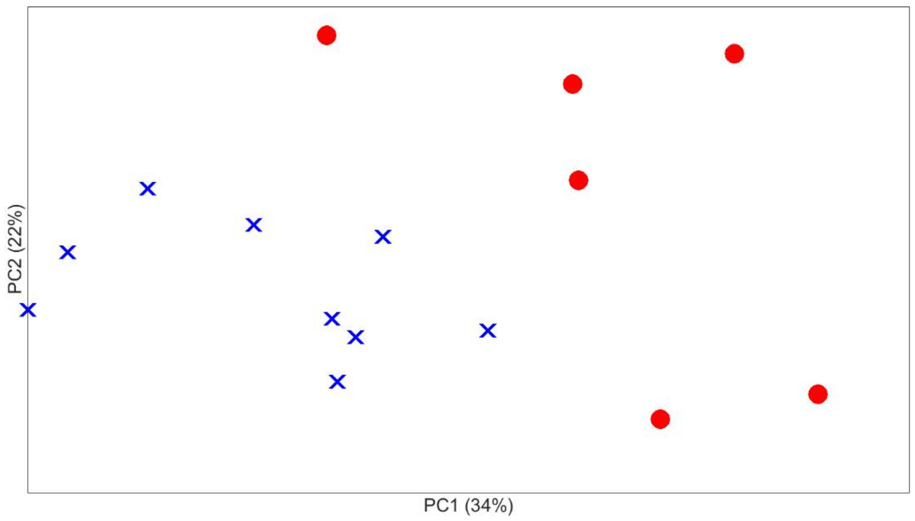

Figure s12: Top PCA score plot of AML data. Each red round represents a control and each blue cross an AML patient.

Multidimensional smoothed histograms; illustrated with the **HIV vaccine trial network data**

Acknowledgement for Paul Eilers, who helped with the multidimensional histogramming.

Introduction

The DAMACY algorithm was also tested on HIV vaccine trial network data (HVTN) of the FLOWCAP II challenge. However, the algorithm only performed with a classification accuracy of 91%. In order to improve the accuracy the base model was built with 3 PCs. The histogram/smoothing algorithm described by Eilers and Goeman was adapted in order to create multidimensional histograms.[2]

HIV vaccine trial network data (HVTN)-In this experiment 48 individuals were given an experimental HIV vaccine. After 10 months, samples were collected and each sample was divided over five vials. In two vials T-cells were challenged with two antigens (ENV-1-PTEG and GAG-1-PTEG). The FLOWCAP II challenge was to discriminate between those two antigens. In another vial an antigen was added, which is known to produce a cytokine response (positive control). The remaining two vials were used as a negative control and nothing was added. The data consists of 5×48 flow cytometry measurements and was gated for only single, live lymphocytes. The following variables were measured in each tube: area and height of forward scatter, area of sideward scatter and 8 surface markers; CD4, ViViD, TNFa, IL4, IFNg, CD8, CD3, IL2 and each sample contained 40,000-350,000 cells.

HVTN adapted methods

The centring and scaling was based on the data of the negative control tubes. The models were built on the training set provided by the FLOWCAP II challenge. A 3-D histogram was built and the number of bins per dimension was 63, so the total number of bins was close to the number of 2-D histograms, *i.e.* 250 thousand bins. Smoothing was performed by first unfolding the histogram $\mathbf{H}_{i_g}$ in one dimension to matrix $\mathbf{C}_{i_g}$ with size $(F_k \times F^{Kbase-1})$ and apply smoothing function seen in equation s5, refold back to original dimension of histogram $\mathbf{H}_{i_g}$ and repeat for the other dimensions until smoothing was applied into every dimension.

(s5) $$(\mathbf{I} + 2\,\lambda\mathbf{D}_1^{\mathbf{T}}\mathbf{D}_1 + \lambda^2\mathbf{D}_2^{\mathbf{T}}\mathbf{D}_2)\,\hat{\mathrm{H}}_{i_g} = \mathbf{H}_{i_g}$$

with $\mathbf{I}$ being the identity matrix, for matrix $\mathbf{D}_1$ holds that $\mathbf{D}_1\mathbf{H} = \Delta\mathbf{H}$ and for matrix $\mathbf{D}_2$ holds that $\mathbf{D}_2\mathbf{H} = \Delta^2\mathbf{H}$, λ is the smoothing factor, this equation results in smoothed histogram $\hat{\mathrm{H}}_{i_g}$ of size $\left(\prod_{k_{base}=1}^{K_{base}} F\right)$.

HVTN results

The HVTN dataset only performed with 91 % accuracy when histograms of the first two PCs were used. At least three PCs were needed to get an accuracy of 100 %. By choosing 3 PCs, 45 % of the total variance is being explained by the PCA model (PC1= 21 %, PC2=13% and PC3=11 %).

After vectorization of the 3-dimensional histograms an OPLS-DA model was built as illustrated in Figure s13. At the left side, the prediction of each sample can be seen. At the right side, three plots show, which area is important for the GAG (blue area) and which is important for the ENV (red area). Only two dimensions can be plotted against each other, therefore each point plotted contains the information of the bins in the other dimension.

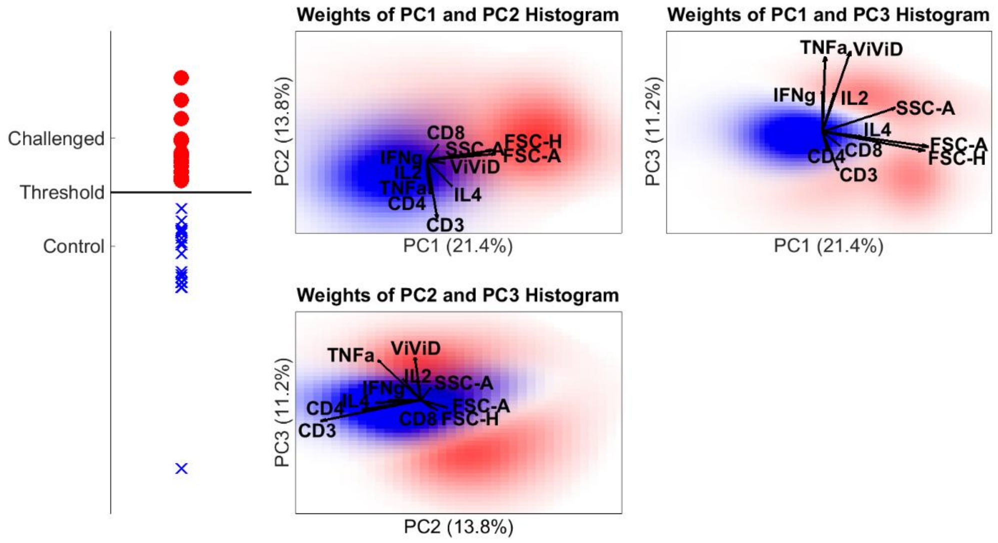

Figure s13: On the left are the individual prediction scores and on the right is the immunological cell map. If the other dimension only contains negative values the bin is colored blue. If the other dimension only contains positive values, the bin is colored red. If the other dimension contains both negative and positive values, the bin is colored purple.

The training set consists of almost 38 million cells, this is probably why three PCs were needed to adequately describe the data and to discriminate between GAG and ENV. The HVTN dataset showed the best results for the first three PCs. Other combinations of two or three principal components would lead to a lower accuracy. The HVTN dataset scored a 100% accuracy, which was comparable with other methods. The DAMACY algorithm works with more PCs, however, the resolutions drops as the number of bins per dimension is lower. Moreover, not a single map is obtained, where the PCs are plotted against each other, but multiple maps for each combination of PCs used. Nevertheless, the DAMACY maps show the marker expression, which other multivariate methods fail to show or need to create a separate figure for each marker.

Analysis of LPS data with ViSNE

Analysis of LPS data with ViSNE

ViSNE analysis was performed on the LPS data using the Matlab GUI cyt. The data was subsampled to contain 1,875 cells in each sample, so the total amount was 30,000 as was optimal according to Amir et. al.[3] First the analysis was performed using the preprocessing in the GUI and the result can be seen in Figure s14. In the lower left corner are mostly cell corresponding the LPS responders and correspond to CD62L-CD16+CD69+CD11b+ cells, which were also found by DAMACY. However, it seems that all cells per individual are cluster together. In the second analysis we performed ViSNE based on our way of preprocessing. In Figure s15 can be seen that no longer cells are clustered together per individual. The same cell cluster of LPS individuals can be found here in the lower right corner. A new cell cluster appears, which contain CD32- cells, but they seem to both present in controls and in LPS.

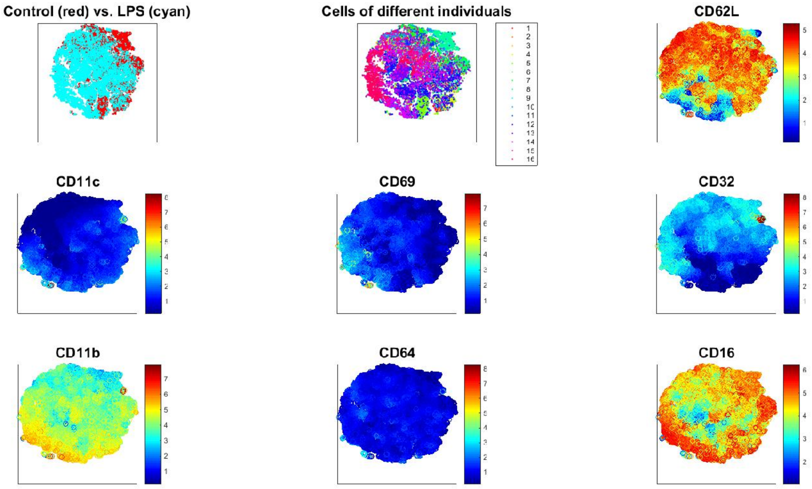

Figure s14: ViSNE analysis performed using the preprocessing built in cyt. In the upper left panel are the cells colored red for controls and cyan for LPS. The cells of other panels are colored based on their logarithmic marker intensity.

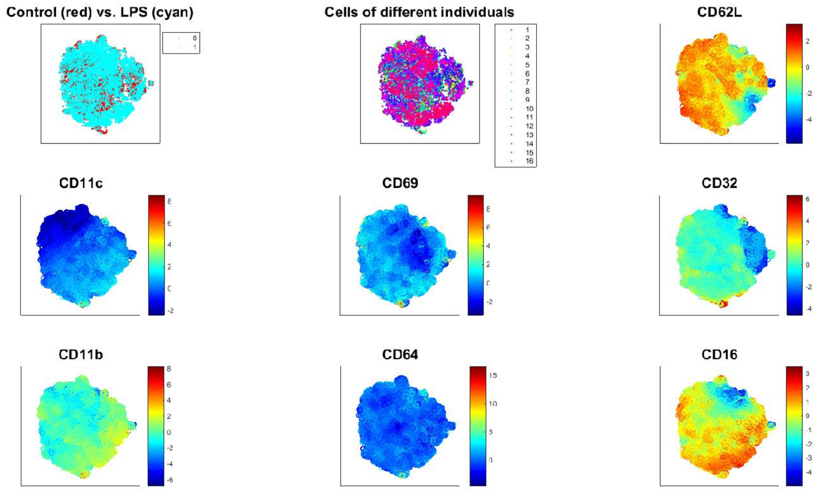

Figure s15: ViSNE analysis performed using the preprocessing built in cyt. In the upper left panel are the cells colored red for controls and cyan for LPS. The cells of other panels are colored based on their logarithmic marker intensity.

Analysis of LPS data with Citrus

Citrus analysis was performed on the LPS data using the R GUI.[4] The data was subsampled to contain 1,875 cells in each sample, so the total amount was 30,000. First the analysis was performed using the pre-processing of the R GUI. The model misclassified 1 sample and the discriminating clusters can be seen in Figure s16. Cluster 29983 and 29992 explain the CD62L+CD16- cells and cluster 29998 the CD62L-CD16+CD11c+CD69+CD11b+ cells. These cells were also found by DAMACY, as seen in Figure s2 and s3. In the second analysis our preprocessing strategy was applied and lead to a perfect classification, however only the CD62L+CD16- population was found.

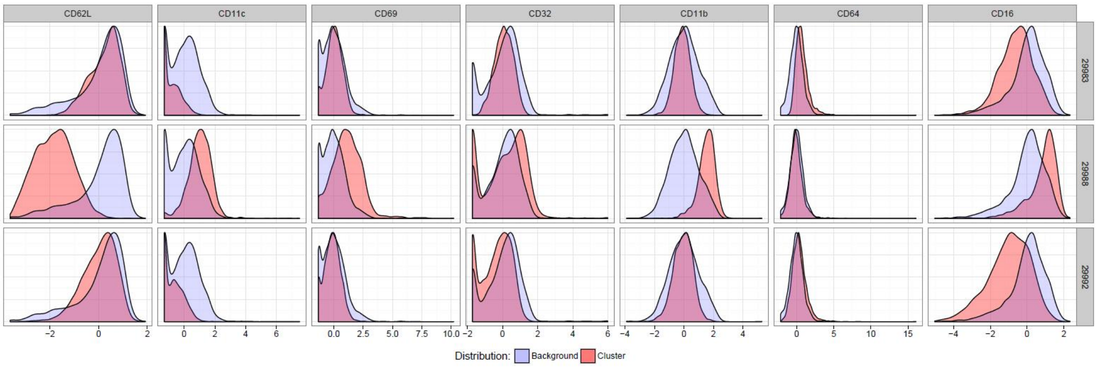

Figure s16: The histograms per marker of the most discriminating clusters.

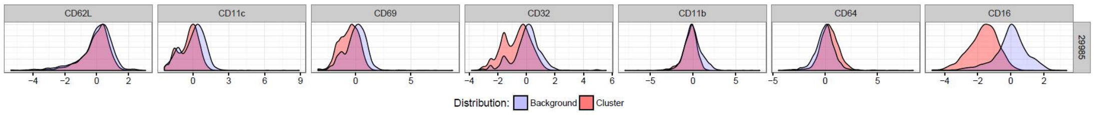

Figure s17: The histograms per marker of the most discriminating clusters.

References

[1] T. Kalina, J. Flores-Montero, V. Van Der Velden, M. Martin-Ayuso, S. Böttcher, M. Ritgen, J. Almeida, L. Lhermitte, V. Asnafi, A. Mendonca, Leukemia, 26 (2012) 1986.
[2] P.H.C. Eilers, J.J. Goeman, Bioinformatics, 20 (2004) 623.
[3] E.-a.D. Amir, K.L. Davis, M.D. Tadmor, E.F. Simonds, J.H. Levine, S.C. Bendall, D.K. Shenfeld, S. Krishnaswamy, G.P. Nolan, D. Pe'er, Nature biotechnology, 31 (2013) 545.
[4] R.V. Bruggner, B. Bodenmiller, D.L. Dill, R.J. Tibshirani, G.P. Nolan, Proceedings of the National Academy of Sciences, 111 (2014) E2770.
